# Rac function is crucial for cell migration but is not required for spreading and focal adhesion formation

**DOI:** 10.1242/jcs.118232

**Published:** 2013-10-15

**Authors:** Anika Steffen, Markus Ladwein, Georgi A. Dimchev, Anke Hein, Lisa Schwenkmezger, Stefan Arens, Kathrin I. Ladwein, J. Margit Holleboom, Florian Schur, J. Victor Small, Janett Schwarz, Ralf Gerhard, Jan Faix, Theresia E. B. Stradal, Cord Brakebusch, Klemens Rottner

**Affiliations:** 1 Institute of Genetics, University of Bonn, Karlrobert-Kreiten Strasse 13, D-53115 Bonn, Germany; 2 Helmholtz Centre for Infection Research (HZI), Inhoffenstrasse 7, D-38124 Braunschweig, Germany; 3 Institute for Molecular Cell Biology, University of Münster, Schlossplatz 5, D-48149 Münster, Germany; 4 Institute of Molecular Biotechnology, Austrian Academy of Sciences, Dr. Bohr-Gasse 3, A-1030 Vienna, Austria; 5 Institute for Toxicology, Hannover Medical School, Carl-Neuberg-Strasse 1, D-30625 Hannover, Germany; 6 Institute for Biophysical Chemistry, Hannover Medical School, Carl-Neuberg-Strasse 1, D-30625 Hannover, Germany; 7 Biomedical Institute, BRIC, University of Copenhagen, DK-2200 Copenhagen, Denmark

**Keywords:** Actin, Rac1, Migration, Adhesion, Lamellipodia, Filopodia, Chemotaxis, CAAX

## Abstract

Cell migration is commonly accompanied by protrusion of membrane ruffles and lamellipodia. In two-dimensional migration, protrusion of these thin sheets of cytoplasm is considered relevant to both exploration of new space and initiation of nascent adhesion to the substratum. Lamellipodium formation can be potently stimulated by Rho GTPases of the Rac subfamily, but also by RhoG or Cdc42. Here we describe viable fibroblast cell lines genetically deficient for Rac1 that lack detectable levels of Rac2 and Rac3. Rac-deficient cells were devoid of apparent lamellipodia, but these structures were restored by expression of either Rac subfamily member, but not by Cdc42 or RhoG. Cells deficient in Rac showed strong reduction in wound closure and random cell migration and a notable loss of sensitivity to a chemotactic gradient. Despite these defects, Rac-deficient cells were able to spread, formed filopodia and established focal adhesions. Spreading in these cells was achieved by the extension of filopodia followed by the advancement of cytoplasmic veils between them. The number and size of focal adhesions as well as their intensity were largely unaffected by genetic removal of *Rac1*. However, Rac deficiency increased the mobility of different components in focal adhesions, potentially explaining how Rac – although not essential – can contribute to focal adhesion assembly. Together, our data demonstrate that Rac signaling is essential for lamellipodium protrusion and for efficient cell migration, but not for spreading or filopodium formation. Our findings also suggest that Rac GTPases are crucial to the establishment or maintenance of polarity in chemotactic migration.

## Introduction

Lamellipodia initiate the migration of various cell types, ranging from fibroblasts and epithelial cells to different types of leukocytes (Small et al., 2002; Renkawitz and Sixt, 2010). Lamellipodia are composed of networks of actin filaments, directly connected in part by branch junctions generated by the Arp2/3 complex (Yang and Svitkina, 2011; Vinzenz et al., 2012). The Arp2/3 complex is activated at the interface between the plasma membrane and growing actin network (Miyoshi et al., 2006; Iwasa and Mullins, 2007; Lai et al., 2008) by Scar/WAVE and associated proteins (Stradal et al., 2001; Stradal and Scita, 2006), now called the WAVE complex (Chen et al., 2010; Derivery and Gautreau, 2010), that accumulates at the tips of lamellipodia (Hahne et al., 2001; Steffen et al., 2004). The pentameric WAVE complex can be directly targeted and activated by the Rho GTPase Rac1 (Ismail et al., 2009; Lebensohn and Kirschner, 2009). Fibroblasts in which Arp2/3 complex expression is stably suppressed (Wu et al., 2012) or ES-cell-derived fibroblastoid cells genetically deficient for the Arp2/3 complex subunit ArpC3 (Suraneni et al., 2012) fail to form lamellipodia, as do cells depleted of WAVE complex components by RNAi (Innocenti et al., 2004; Steffen et al., 2004; Steffen et al., 2006; Nicholson-Dykstra and Higgs, 2008), confirming the importance of WAVE to Arp2/3 complex signaling for lamellipodial protrusion. However, the impact of lamellipodium suppression on migration efficiency remained controversial (Suraneni et al., 2012; Wu et al., 2012).

The family of Rho GTPases comprises 20 members in mammals (Heasman and Ridley, 2008; Ladwein and Rottner, 2008), overexpression of most of which drives major reorganizations of the actin cytoskeleton (Aspenström et al., 2004). Although Rac proteins such as Rac1 are known to induce formation of lamellipodia and membrane ruffles (Ridley et al., 1992; Nobes and Hall, 1995), other Rho proteins such as the Rac-related RhoG or Cdc42 can also trigger these structures (Nobes and Hall, 1995; Aspenström et al., 2004). Analyses of migratory performance of primary fibroblasts depleted of Rho proteins by RNAi have recently indicated multiple, redundant pathways operating downstream of Cdc42, Rac proteins and RhoG (Monypenny et al., 2009). Although biochemical connections established potential convergence of signaling on Rac, both downstream of Cdc42 (Baird et al., 2005; Nishimura et al., 2005) and RhoG (Katoh and Negishi, 2003), it remained unclear whether Cdc42 or RhoG could also induce cytoskeletal rearrangements by bypassing Rac GTPases. For instance, although a direct interaction of the WAVE complex with RhoG-GTP had previously been proposed (Ridley, 2006), binding of this GTPase to the WAVE complex was reported to be rather weak and nucleotide independent (Meller et al., 2008). Furthermore, it could not be formally excluded that Cdc42 can drive lamellipodium formation independent of Rac.

Conditional Rac1 alleles in fibroblasts were previously deleted by Cre recombinase delivered through adenovirus (Guo et al., 2006) or a cell-permeable HIV–TAT fusion (Vidali et al., 2006). Interestingly, although Vidali and colleagues found only moderate effects on migration efficiency, cell spreading and focal adhesion formation, Guo et al. concluded Rac1 to be essential for focal adhesion and stress fiber assembly (Guo et al., 2006). More recently, modest defects in migration, adhesion site and stress fiber formation were also observed in primary fibroblasts isolated after tamoxifen-mediated Rac1 deletion in mice (Liu et al., 2009). In spite of reasonably efficient Rac1 gene deletion in these populations, no individual clones and thus permanent *Rac1*^−/−^ cell lines were developed, precluding faithful analysis of the consequences of Rac1 gene deletion at the single cell level. The Rac-deficient cell lines developed for our study avoid the possibility that variability in phenotypes could derive from incomplete Rac1 removal.

## Results

### Generation of permanent *Rac1*^−/−^ fibroblast cell lines

**Fig. 1.**
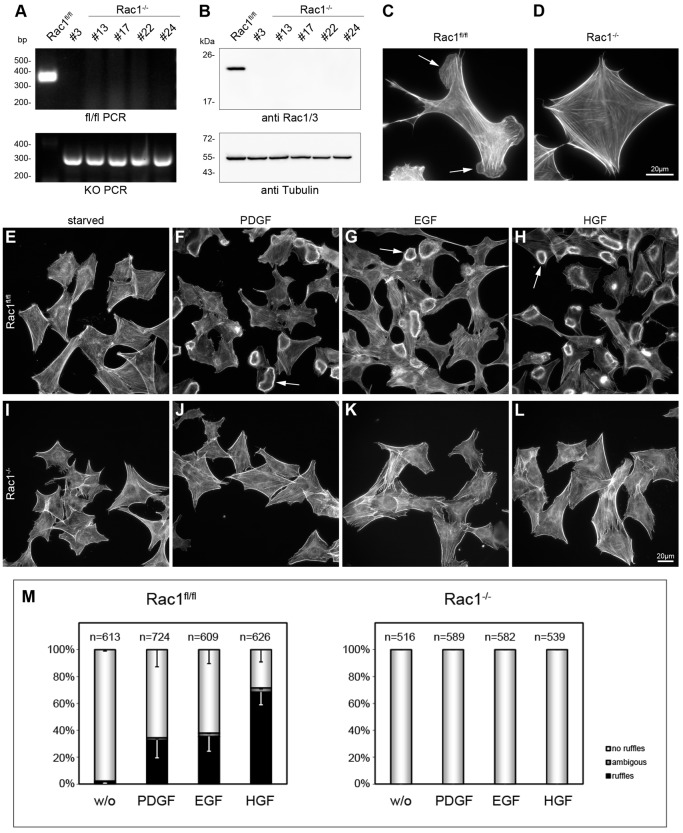
**Rac1-deficient MEFs are unable to form lamellipodia and ruffles.** (A) Genotyping of *Rac1^fl/fl^* cells and individual *Rac1*^−/−^ clones for genotypes as indicated. (B) Western blot of *Rac1^fl/fl^* and individual *Rac1*^−/−^ clones with Rac1/3 antibody (upper panel) and tubulin antibody (lower panel). (C,D) Phalloidin staining of *Rac1^fl/fl^* (C) and *Rac1*^−/−^ MEFs (D). (E–L) Growth factor stimulation. *Rac1^fl/fl^* MEFs (E–H) and *Rac1*^−/−^ MEFs (I–L) were either starved (E,I), or stimulated with PDGF (F,J), EGF (G,K) or HGF (H,L). (M) Quantification of growth factor stimulations. Data were collected from three independent experiments and are expressed as means ± s.e.m.; *n* = total number of cells analyzed.

Rac1 was deleted in *Rac1^fl/fl^* mouse embryonic fibroblasts (MEFs) by Cre recombinase. Individual clones were isolated and genotyped for the presence of excised and floxed alleles. Rac1 alleles harboring the respective deletion in exon 3 was detected in all clones obtained after isolation and further expansion (more than a dozen; for a selection of clones see Fig. 1A). Loss of Rac1 protein was also confirmed by western blotting (Fig. 1B), employing an antibody that recognizes Rac1 and Rac3 equally well (supplementary material Fig. S1A). Rac3 expression is restricted to specific stages of brain development (Bolis et al., 2003; Corbetta et al., 2005) and Rac2 expression is confined to hematopoietic cells (Didsbury et al., 1989). Although microarray analyses indicated increased *Rac3* mRNA in *Rac1*^−/−^ clones selected for further analyses (see below), absolute levels in these clones hardly exceeded threshold counts (unpublished data), and Rac3 protein remained undetectable by western blotting, as was the case for Rac1 and Rac2 (Fig. 1B, supplementary material Fig. S1A–C). These cell lines thus constitute the first, virtually Rac-free fibroblastoid cell system. By sequence analysis, it was determined that RhoG belongs to the Rac family, bearing 72% identity to Rac1 (Heasman and Ridley, 2008). Interestingly, Rac1-deficient cells showed unchanged RhoG transcript (unpublished data) and protein levels (supplementary material Fig. S1D). For subsequent experiments, five Rac1-deficient clones were selected, based on relative comparability of growth rates, and termed #3, #13, #17, #22 and #24 (Fig. 1A,B). In order to minimize potential clonal variability of the studied phenotypes, individual *Rac1*^−/−^ clones were maintained separately and pooled prior to each experiment. Surprisingly, all *Rac1*^−/−^ clones were able to divide continuously over years, albeit at a much slower propagation rate than control MEFs (supplementary material Fig. S1E), showing that Rac1 is not absolutely essential for proliferation.

### Rac1 is essential for lamellipodia and ruffle formation in fibroblasts

To confirm the crucial function of Rac GTPases in the regulation of specific actin structures, Rac1-deficient MEFs and their parental, floxed counterparts were seeded on fibronectin and stained for the actin cytoskeleton. As expected, Rac1-expressing control fibroblasts formed spontaneous lamellipodia at the cell periphery (Fig. 1C), whereas Rac1-deficient MEFs appeared completely devoid of these structures (Fig. 1D), confirming previous observations (Guo et al., 2006; Vidali et al., 2006). The majority of Rac1-deficient cells had lost the slightly elongated and frequently polarized morphology typical of fibroblasts, and developed a starfish-like architecture instead, displaying prominent actin bundles between peripheral attachment points. Otherwise, the overall stress fiber pattern appeared comparable in both cell types (Fig. 1C,D), at variance with previous observations (Guo et al., 2006; Liu et al., 2009). Furthermore, we did not observe noticeable blebbing in these cells lacking lamellipodia, as was previously associated with Rac1 loss of function (Vidali et al., 2006) and inhibition of Arp2/3-mediated lamellipodium formation (Bergert et al., 2012). Filopodium formation was also commonly observed (see below), indicating that distinct protrusion types, lamellipodia, filopodia and blebs may be interdependent, to certain extents under specific conditions, yet are mechanistically separable.

To explore Rac functions in stimulated actin cytoskeletal rearrangements, we employed treatments with various growth factors. *Rac1^fl/fl^* cells responded within minutes to PDGF, EGF and HGF addition with the formation of prominent dorsal ruffles (Fig. 1F–H) but few peripheral ruffles (unpublished data). In contrast, dorsal ruffle formation was entirely abolished in Rac1-deficient fibroblasts (Fig. 1J–L). The frequency of dorsal ruffle formation in Rac1 control cells was highest after HGF treatment (68%), whereas 33% and 35% of Rac1 control cells showed ruffles after PDGF and EGF treatment, respectively. We failed to detect a single Rac1-deficient cell capable of dorsal ruffling upon treatment with any one of the different growth factors (1710 cells analyzed in total, see quantification in Fig. 1M). These data strongly suggest an essential role for Rac proteins in growth-factor-induced membrane ruffling as well as lamellipodium formation stimulated, for example, in response to extracellular matrices such as fibronectin.

### All Rac proteins restore lamellipodium formation and interact with the WAVE complex

**Fig. 2.**
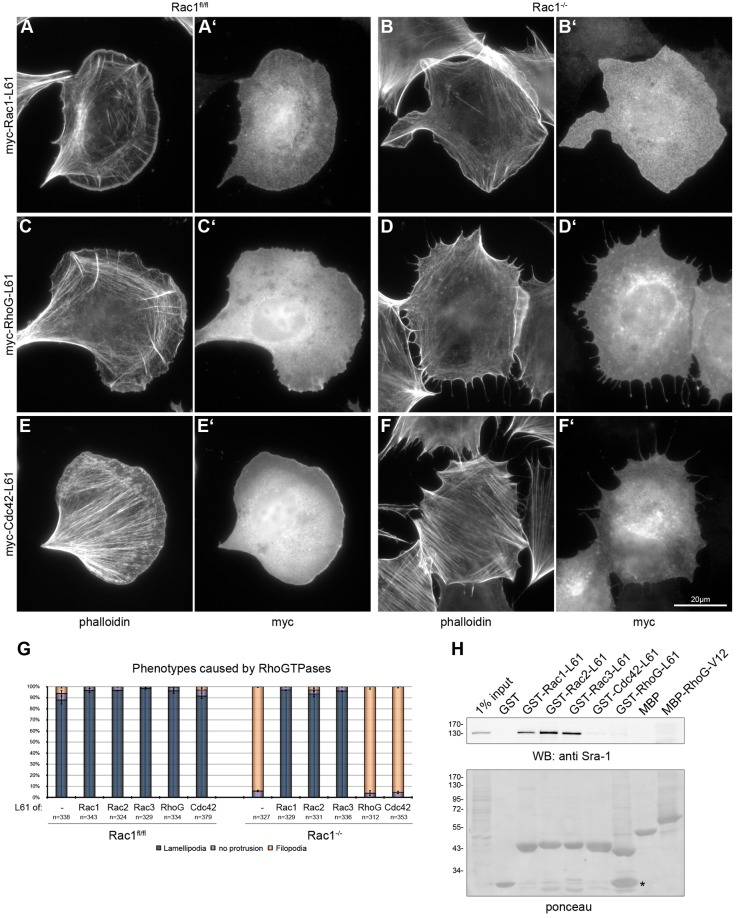
**Rac1, Rac2 and Rac3 restore lamellipodia and interact with the WAVE complex, but not RhoG and Cdc42.** (A–F′) Expression of constitutively active Rho GTPases in *Rac1^fl/fl^* and *Rac1*^−/−^ cells. *Rac1^fl/fl^* (A,A′,C,C′,E,E′) and *Rac1*^−/−^ (B,B′,D,D′,F,F′) MEFs were transfected with myc-tagged Rac1-L61 (A–B′), RhoG-L61 (C–D′) and Cdc42-L61 (E–F′), fixed and stained with phalloidin (A,B,C,D) and anti-myc (A′,B′,C′,D′). Note the absence of lamellipodia in D and E as opposed to A, B, C and E. (G) Quantification of phenotypes caused by expression of different Rho GTPases. *Rac1^fl/fl^* and *Rac1*^−/−^ MEFs without overexpression of any Rho GTPase (–) or transfected with myc-tagged versions of constitutively active (L61) GTPases as indicated were evaluated. Cells were first scored for the presence of lamellipodia (blue). In case no lamellipodia were present, the presence of filopodia was assessed (light orange), and in case neither lamellipodia nor filopodia were detectable, cells were scored as ‘no protrusion’ (purple). Data were collected from three independent experiments and are expressed as means ± s.e.m.; *n* = total number of cells analyzed. (H) WAVE complex interacts with Rac1, Rac2 and Rac3, but not RhoG and Cdc42. Pull down of recombinantly expressed Rho GTPases and controls as indicated shows Sra-1 binding to Rac1, Rac2 and Rac3 (upper panel). The lower panel shows input of recombinant proteins. As GST–RhoG-L61 was proteolytically cleaved in part (asterisk), MBP-tagged RhoG-V12 was used as confirmation.

To confirm that the absence of lamellipodium formation in Rac-deficient cells is due solely to the absence of a Rac GTPase, and not to secondary events, we ectopically expressed constitutively active variants of Rac1, 2 or 3 as well as active forms of Cdc42 and RhoG. This approach also allowed a direct comparison of the efficiency of lamellipodium induction by distinct Rac proteins in the same cell type. As described in the initial characterization of Rac1 function in fibroblasts (Ridley et al., 1992), expression of a constitutively active Rac1, Rac1-L61, induced lamellipodia in control fibroblasts (Fig. 2A,A′). This phenotype was virtually indistinguishable from that of cells lacking endogenous Rac1 (Fig. 2B,B′), indicating full restoration of Rac1 gene loss of function by ectopic Rac1 re-expression (for overview images see supplementary material Fig. S2). Microinjection of constitutively active Rac1-L61 protein caused abrupt induction of lamellipodia (supplementary material Movie 1 and supplementary material Fig. S3). These data confirmed the presence of a dormant lamellipodial machinery readily receptive to activation by Rac1. Moreover, Rac1 protein harboring an alternative, constitutively active variant (Rac1-V12) as well as wild-type Rac1 had comparable effects (supplementary material Movies 2 and 3; Fig. S3), indicating potential GEF-mediated Rac GTP-loading upon injection of the wild-type protein. Furthermore, constitutively active Rac2 or Rac3 had effects identical to Rac1-L61 (supplementary material Fig. S4B,D; for quantifications see Fig. 2G).

In control cells, Rac2, Rac3 (supplementary material Fig. S4A,C), RhoG and Cdc42 (Fig. 2C,E) triggered lamellipodium formation, with efficiencies comparable to that of Rac1 (>90%, Fig. 2G) and similar to previous studies (Aspenström et al., 2004). However, unlike Rac2-L61 and Rac3-L61, active RhoG and Cdc42 completely failed to generate Rac1-deficient cells displaying lamellipodia (0% of *Rac1*^−/−^ cells with lamellipodia; Fig. 2D,F,G; supplementary material Fig. S4).

Aside from the lack of apparent lamellipodia-like structures, Rac1-deficient fibroblasts frequently projected extensions at their periphery (Fig. 2D,F,G) that exhibited the dynamics of bona-fide filopodia, including protrusion parallel to the substrate as well as frequent up-lifting and kinking (supplementary material Movies 1–3). These observations are consistent with the formation of filopodia in cells devoid of lamellipodia, as proposed in early RNAi studies (Steffen et al., 2006; Gomez et al., 2007; Nicholson-Dykstra and Higgs, 2008), and confirmed more recently using stable Arp2/3 complex suppression or genetic deletion of the Arp2/3 complex subunit ArpC3 (Suraneni et al., 2012; Wu et al., 2012).

Together, these results strongly argue for a redundant function of different Rac proteins in lamellipodium formation, with RhoG and Cdc42 acting upstream of the Rac pathway. Our data also suggest that Rac proteins are equally capable of interacting with the downstream lamellipodial machinery. To investigate this pathway biochemically, we tested the interaction of individual GTPases with the WAVE complex. The pentameric WAVE complex, comprising WAVE1/2, HSPC300, Abi1/2, Nap1 and Sra-1/PIR121 is essential for lamellipodium protrusion (Stradal et al., 2004; Takenawa and Suetsugu, 2007). Sra-1 was identified as a direct binding partner of active Rac1 (Kobayashi et al., 1998), and shown to bind to RhoG at best weakly and independent of nucleotide loading (Meller et al., 2008), but WAVE complex association with other Rac proteins has not yet been analyzed. Recombinant Rac1, Rac2 and Rac3 interacted with the WAVE complex as evidenced by binding of Sra-1 (Fig. 2H). In contrast, we failed to detect specific interactions of Sra-1 with either Cdc42 or RhoG. In conclusion, Cdc42 and RhoG probably fail to induce lamellipodia in the absence of endogenous or ectopic Rac proteins because of their inability to connect to WAVE.

### Post-translational prenylation of Rac1 is not essential for signaling to actin reorganization

**Fig. 3.**
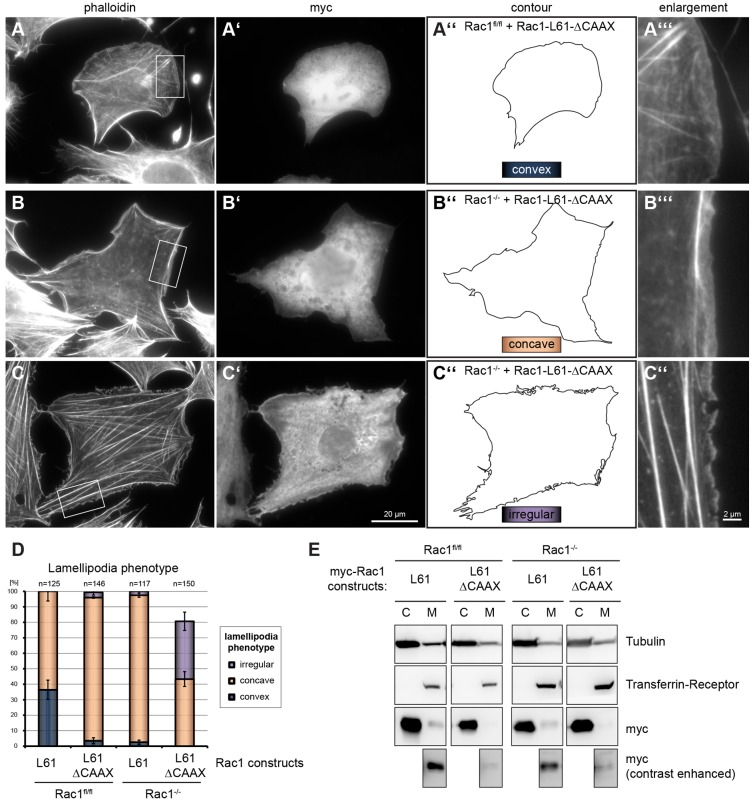
**Rac1-ΔCAAX is capable of robust but not full rescue of lamellipodium formation.** *Rac1^fl/fl^* (A) and *Rac1*^−/−^ (B,C) cells expressing myc-Rac1-L61-ΔCAAX form lamellipodia with distinct phenotypes. For quantification of robustness of lamellipodium formation, categories were assigned according to their contours as follows: regular lamellipodia associated with convex cell outline (see A″), regular lamellipodia associated with concave cell outline (B″) and small, irregular lamellipodia (C″). Cells were transfected with myc-Rac1-L61 (not shown, see also Fig. 2) and myc-Rac1-L61-ΔCAAX and stained with phalloidin (A,B,C) and anti-myc (A′,B′,C′) to identify transfected cells. Enlargements of the actin cytoskeleton at the cell periphery of regions boxed in A–C are shown in A″,B″,C″. Scale bars in C′ (for A,A′,B,B′,C) and C″ (A″,B″). (D) Quantification of lamellipodial phenotype. *Rac1^fl/fl^* and *Rac1*^−/−^ MEFs expressing Rac1 constructs as indicated were assessed for the presence of convex, concave or irregular lamellipodia. Data are from three independent experiments and expressed as means ± s.e.m.; *n* = total number of cells analyzed. (E) *Rac1^fl/fl^* and *Rac1*^−/−^ cells transfected with myc-tagged Rac1 constructs, as indicated, were separated into cytosol (C) and membrane (M) fractions. Samples were probed with antibodies as indicated. Anti-myc-probed membrane fractions are additionally shown with contrast enhancement (bottom panels).

Ras- and Rho-family GTPases harbor the common, C-terminal CAAX motif that is modified by the polyisoprene lipid geranylgeranyl in case of Rac1. Prenylation of the C-termini of Rho GTPases has long been considered essential for their membrane association and thus correct biological function (Hori et al., 1991; Solski et al., 2002). For instance, the YopT effector from pathogenic *Yersinia* strains is a cysteine protease that cleaves directly upstream of the modified cysteine (Shao et al., 2003), thereby releasing the GTPase from the membrane and inducing its passage to the nucleus (Wong and Isberg, 2005). C-terminal prenylation of Rac1 was also concluded to be a pre-requisite for its palmitoylation on cysteine 178, recently implicated in proper plasma membrane partitioning and Rac1-mediated actin remodeling (Navarro-Lérida et al., 2012). However, genetic deletion of geranylgeranyltransferase type I (GGTase I) in fibroblasts and macrophages recently showed Rho-GTPase prenylation to have functions beyond solely being an essential prerequisite for membrane positioning and activation (Philips, 2011). Indeed, GGTase-I-deficient macrophages have strongly increased rather than decreased levels of active Rho, Rac and Cdc42 (Khan et al., 2011). The virtual absence of endogenous Rac GTPases in our permanent cells lines allowed comparison of the efficiency of actin remodeling by ectopic Rac1 harboring or lacking the C-terminal CAAX box (CLLL). Surprisingly, *Rac1*^−/−^ cells expressing Rac1 lacking the CLLL sequence (ΔCAAX) formed lamellipodia and membrane ruffles quite robustly, although thorough quantification revealed modest defects in actin remodeling efficiency. The penetrance of lamellipodium induction was classified into three categories as shown in Fig. 3A–C. The strongest phenotype was represented by cells with large, convex lamellipodia (Fig. 3A) with smooth edges, formed in 36% of *Rac1^fl/fl^* cells expressing constitutively active Rac1, but was not in the same cells expressing the ΔCAAX version (Fig. 3D). A slightly less prominent Rac response was represented by concave-shaped lamellipodial actin networks (Fig. 3B), and the weakest form of reconstitution of Rac activity was morphologically characterized by lamellipodia with irregular, frayed edges (Fig. 3C). Approximately half of the *Rac1*^−/−^ cells expressing the ΔCAAX variant showed the latter phenotype, which was not seen in *Rac1*^−/−^ cells transfected with constitutively active, full-length Rac1. Direct comparison of control and *Rac1*^−/−^ cells ectopically expressing Rac1-L61 and the Rac1-ΔCAAX variant reveals that the presence of endogenous Rac1 significantly contributes to phenotypic outcome. In summary, this analysis established that although helpful for efficient signaling to actin remodeling, prenylation of Rac1 and probably the subsequent post-translational modifications such as palmitoylation are not essential for inducing actin polymerization in lamellipodia. Finally, Rac1-ΔCAAX-expressing *Rac1*^−/−^ cells had lamellipodia with WAVE complex at their tips (not shown), consistent with WAVE/Arp2/3 being essential for lamellipodia. To biochemically compare the subcellular location of the myc-tagged Rac1 variants used, transfected *Rac1^fl/fl^* cells were separated into cytosol and membrane fraction, and analyzed by western blotting. Rac1-ΔCAAX was clearly reduced in the membrane fraction (Fig. 3E), indicating compromised plasma membrane association of Rac1-ΔCAAX. To what extent the polybasic region N-terminal to CLLL in Rac1 may contribute to residual plasma membrane association (Philips, 2011), and whether the WAVE complex might at least partly be activated in the cytosol prior to lamellipodial targeting, will be addressed in future studies.

### Migration capacity is dramatically impaired in Rac1-deficient fibroblasts

**Fig. 4.**
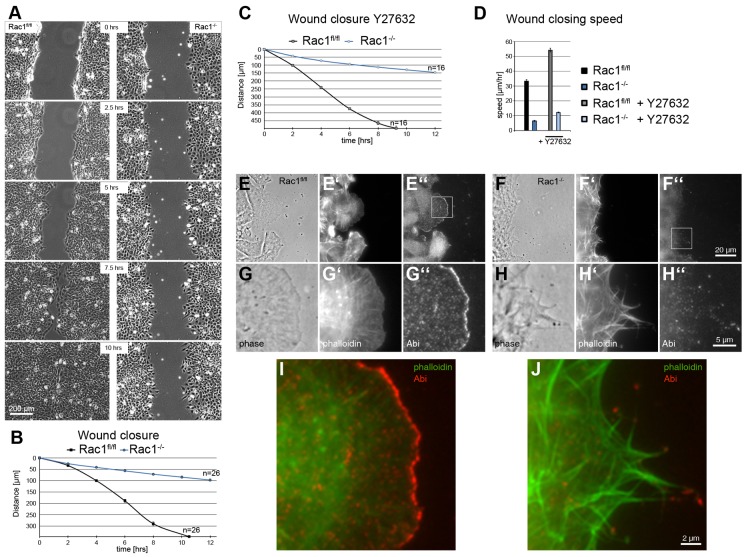
**Migration capacity is strongly reduced in Rac1-deficient MEFs.** (A) Selected frames from wound healing movies of control (*Rac1^fl/fl^*) and *Rac1*^−/−^ cells. (B) Average wound closure for each cell type over time. *Rac1*^−/−^ cells are not able to close the wound after 20 hours (see supplementary material Fig. S5). Data were collected from three independent experiments; *n* = total number of movies analyzed. Error bars indicate ± s.e.m. (C) Average wound closure for each cell type treated with Rho kinase inhibitor Y27632 over time. Data were collected from two independent experiments; *n* = total number of movies analyzed. Error bars represent ± s.e.m. (D) Wound closing speed of *Rac1^fl/fl^* and *Rac1*^−/−^ cells with or without Y27632 treatment. Note that Rho kinase inhibition increases the wound closure speed in both cell types to the same extent. Error bars indicate ± s.e.m. (E–J) The leading front of *Rac1*^−/−^ cells facing the wound area is devoid of lamellipodia but has numerous filopodia. *Rac1^fl/fl^* (E–E″,G–G″,I) and *Rac1*^−/−^ cells (F–F″,H–H″,J) 4 hours after wounding were stained with phalloidin (E′,F′,G′,H′) and anti-Abi (E″,F″,G″,H″). (E,F,G,H) phase contrast, (I,J) merged images.

Owing to the strong phenotype in lamellipodium formation in the absence of Rac GTPases, we investigated how this might coincide with the efficiency of cell migration. Wounds induced in cell monolayers of *Rac1*^−/−^ cells failed to close after 20 hours (supplementary material Fig. S5) whereas closure was achieved after an average of 10.5 hours with *Rac1^fl/fl^* cells (Fig. 4A,B, see also supplementary material Movie 4). Migration rates were found to be reduced more than fivefold in Rac1-deficient cells (33.4 µm/hour and 6.5 µm/hour for *Rac1^fl/fl^* and *Rac1*^−/−^ fibroblasts, respectively; Fig. 4D). However, migration in these conditions was not entirely eliminated, so we examined whether migration into a wound in the absence of Rac could involve myosin-II-based contraction. To address this, we inhibited the RhoA effector Rho-kinase (ROCK), a major target of signaling to myosin-II-mediated contractility. To our surprise, ROCK inhibition by Y27632 increased migration speed of both, *Rac1^fl/fl^* and *Rac1*^−/−^ cells, to 54 µm/hour and 12 µm/hour, respectively. These data suggested that myosin activity is not crucial for the residual motility observed in the absence of Rac and that ROCK-inhibitor-mediated increase of migration does not require Rac function. Since *Rac1*^−/−^ fibroblasts lacked spontaneous lamellipodia, we sought to investigate the actin cytoskeleton of these cells at the wound edge, in particular, since migration was strongly reduced but not abolished. To explore whether *Rac1*^−/−^ cells employed lamellipodia for migration, we stained cells for F-actin and the WAVE complex. As opposed to control cells, which had prominent Abi staining at the tips of lamellipodia formed at the wound edge (Fig. 4G,G′,G″,I), the cell periphery of *Rac1*^−/−^ cells was essentially unstained, except for dot-like Abi accumulations at filopodium tips (Fig. 4H,H′,H″,J). Filopodia frequently projected into the wound area, which suggests a filopodia-driven mechanism of migration, operating – at least in part – under these conditions.

### Rac signaling is essential for chemotaxis

**Fig. 5.**
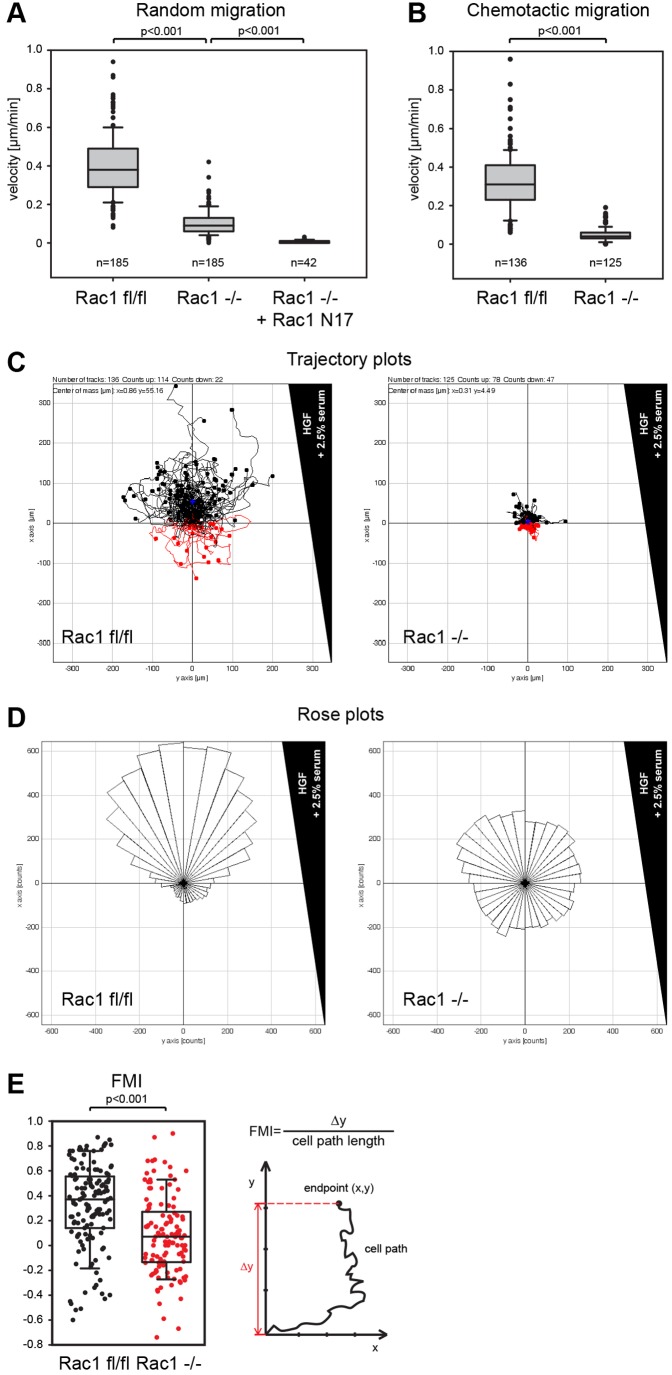
**Random migration and chemotaxis are abrogated in Rac1-deficient MEFs.** (A) Random migration of *Rac1^fl/fl^*, *Rac1*^−/−^ and *Rac1*^−/−^ cells expressing dominant-negative Rac1-N17 (medians 0.380, 0.090, 0.000, respectively). Data sets are from three independent experiments, *n* = total number of cells analyzed. (B) Chemotactic migration of *Rac1^fl/fl^* and *Rac1*^−/−^ cells towards a gradient of 2.5% serum and 100 ng/ml HGF (medians 0.310 and 0.040, respectively). Data sets are from three independent experiments, *n* = total number of cells analyzed. (C) Trajectory plots of *Rac1^fl/fl^* and *Rac1*^−/−^ cells in the chemotaxis assay show all individual cell paths. Migration paths towards the HGF-gradient are colored black, migration paths away from the growth factor source are colored red. (D) Rose plots of 10° segments showing the frequency of a given direction of migration paths during chemotaxis. (E) Forward migration index (FMI) of *Rac1^fl/fl^* and *Rac1*^−/−^ cells during chemotaxis (medians 0.370 and 0.070, respectively). Box and whiskers plots show medians, 10th, 25th, 75th and 90th percentiles and dots show individual data points.

The strong reduction in wound-healing migration could derive both from migration defects and problems with initiation or maintenance of polarity or both. To distinguish between these possibilities, we performed additional assays. In random migration assays, *Rac1*^−/−^ cells showed a fourfold reduction of migration velocity, suggesting that the reduction in wound healing migration was mostly caused by migratory defects (Fig. 5A). In line with previous conclusions (Vidali et al., 2006), ectopic expression of dominant-negative Rac1 in *Rac1*^−/−^ cells eliminated migration, establishing that effects exerted by Rac1-N17 cannot solely be explained by interference with Rac functions (Fig. 5A).

Next we explored the migration capacity towards chemotactic stimuli. To set up chemotaxis, we used 2.5% serum in combination with hepatocyte growth factor, as induction of ruffling in *Rac1^fl/fl^* cells was most robust with this growth factor (Fig. 1). Interestingly, migration velocity upon Rac deletion in this assay was even more drastically reduced than in wound healing or random migration (Fig. 5B,C), arguing for directionality-dependent, additional defects. Indeed, when a bias for a given direction of migration was identified by Rose plots (Fig. 5D), the data revealed a prominent additional defect, as *Rac1*^−/−^ cells almost entirely lacked an increase of path frequency towards the gradient. This difference is also reflected by the forward migration index, the median of which approached zero (essentially no directional migration) in *Rac1*^−/−^ cells. These data strongly suggest that the migration defects observed in *Rac1*^−/−^ cells are accompanied by an additional, independent lack of ability of these cells to migrate towards chemotactic stimuli.

### Rac is not required for cell spreading

**Fig. 6.**
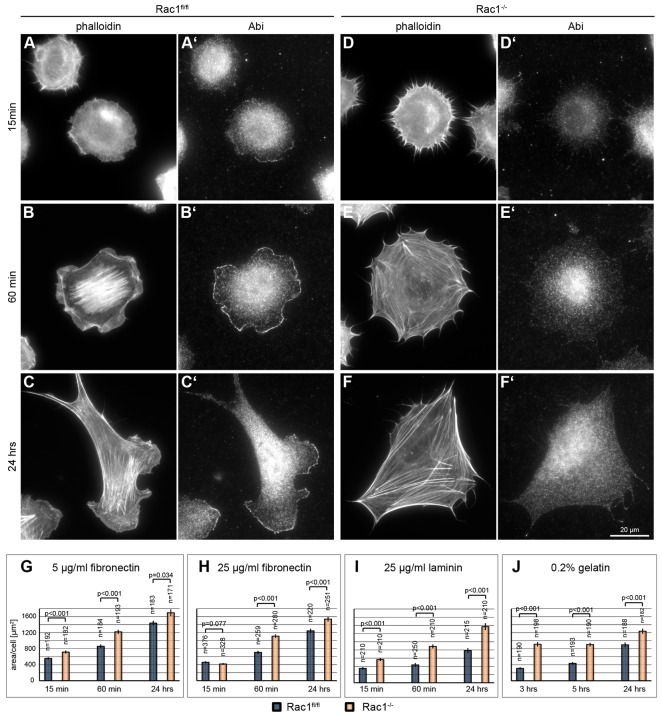
**Rac1 is not required for cell spreading.** (A–F′) Immunofluorescence of spreading cells. *Rac1^fl/fl^* (A–C′) and *Rac1*^−/−^ cells (D–F′) were stained with phalloidin (A,B,C,D,E,F) and anti-Abi (A′,B′,C′,D′,E′,F′) 15 minutes (A,A′,D,D′), 60 minutes (B,B′,E,E″) and 24 hours (C,C′,F,F″) after plating on 25 µg/ml fibronectin. Note that localization of Abi at the lamellipodium tip, prominent in B′ and C′, is absent in *Rac1*^−/−^ cells. (G–J) Quantification of the spreading area on different fibronectin concentrations or extracellular matrices. *Rac1^fl/fl^* and *Rac1*^−/−^ MEFs were plated on 5 µg/ml fibronectin (G), 25 µg/ml fibronectin (H), 25 µg/ml laminin (I) or 0.2% gelatin (J) as detailed in the Materials and Methods. All datasets are from three independent experiments. Error bars indicate the s.e.m.; *n* = total number of cells analyzed.

Rac GTPases have previously been implicated in both lamellipodium formation and the regulation of cell spreading (Guo et al., 2006; Vidali et al., 2006; Liu et al., 2009). Since loss of lamellipodia correlated well with a strong reduction in directed or random cell migration, we sought to explore the effects of Rac loss of function on spreading. Initial observations during clonal expansion and maintenance of Rac-deficient cells indicated only minor problems with adhesion to or spreading on tissue-culture dishes. To address this more directly, control and knockout fibroblasts were allowed to spread on fibronectin (25 µg/ml) and analyzed after different times. Again, F-actin staining revealed the virtual absence of lamellipodia in *Rac1*^−/−^ cells (Fig. 6D–F), confirmed by immunostaining for Abi (Fig. 6D′,E′,F′). In contrast, control cells had prominent lamellipodia after 24 hours on fibronectin (Fig. 6C), with Abi proteins at their tips (Fig. 6C′). Lamellipodium formation was also observed at earlier time points, (Fig. 6A,A′,B,B′), albeit less frequently. Remarkably, quantification of cell areas at different times after initiation of spreading revealed no defects in the absence of Rac and lamellipodia (Fig. 6H). Indeed, when quantifying spreading efficiency on coverslips coated with low concentrations of fibronectin (5 µg/ml; Fig. 6G) as well as on laminin (Fig. 6I) or gelatin (Fig. 6J), *Rac1*^−/−^ cells clearly covered an even larger cell area than controls. These data show, for the first time, that Rac-GTPase function and thus lamellipodia are dispensable for spreading, independent of engagement with a specific integrin receptor, although Rac function had frequently been implicated in spreading previously (Guo et al., 2006; Vidali et al., 2006; Liu et al., 2009). Our data are also at variance with those from cells with reduced Arp2/3 complex expression (Wu et al., 2012), although the defect observed upon genetic removal of the Arp2/3 complex subunit ArpC3 was much less pronounced. Although the reasons for these discrepancies are currently unclear, these experiments show that spreading and lamellipodium formation or migration efficiency can be functionally uncoupled.

### Rac is not required for focal adhesion and filopodium formation

Since spreading of *Rac1*^−/−^ fibroblasts appeared increased rather than decreased compared with controls, we wondered how focal adhesions are assembled and turned over during this process. Formation of nascent adhesions is frequently described to occur below lamellipodia, close to the protruding front (Choi et al., 2008), so we were curious to explore adhesion formation in these cells spreading without lamellipodia.

**Fig. 7.**
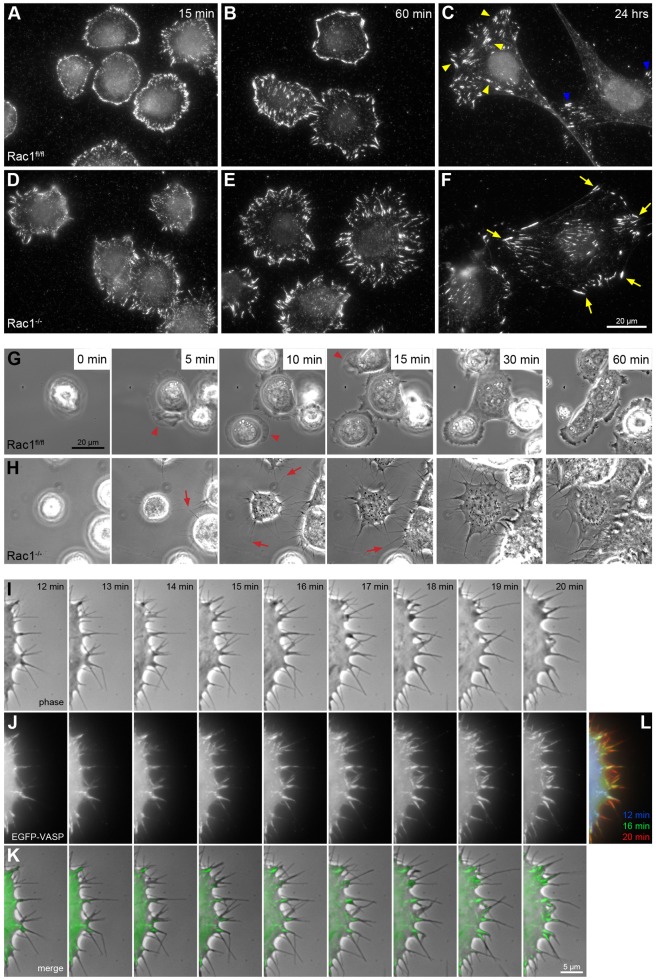
**Rac1 is not essential for focal adhesion and filopodium formation.** (A–F) Vinculin staining of *Rac1^fl/fl^* (A–C) and *Rac1*^−/−^ (D–F) cells 15 minutes (A,D), 60 minutes (B,E) and 24 hours (C,F) after plating. After 24 hours, the distribution of focal adhesions is more variable between cells than at earlier time points. Some cells show focal adhesions evenly distributed under the cell (yellow arrowheads in C), while some display focal adhesions mainly at peripheral attachment points (blue arrowheads in C). As opposed to controls after 24 hours of spreading, most peripheral attachment points in *Rac1*^−/−^ cells appeared equidistant from the nucleus, indicating lack of polarization (yellow arrows in F). (G,H) *Rac1*^−/−^ MEFs spread by employing filopodia. Selected frames of phase-contrast movies of *Rac1^fl/fl^* (G) and *Rac1*^−/−^ cells (H) acquired during spreading. Arrowheads point to lamellipodia, arrows point to filopodia. (I–K) Focal adhesions are formed at the base of filopodia in *Rac1*^−/−^ cells. Selected phase-contrast (I) and green epifluorescence channel (J) frames of *Rac1*^−/−^ cells expressing GFP–VASP, show accumulation of VASP in focal adhesions formed at the base of protruding filopodia. (K) shows merged phase contrast (gray) and GFP–VASP (green) frames. (L) Maturation of focal adhesions over time is shown in a merge of GFP-VASP images from three different time points. Blue corresponds to GFP-VASP localization after 12 minutes, green corresponds to 16 minutes and red to 20 minutes.

*Rac1^fl/fl^* and *Rac1*^−/−^ cells were stained for the focal adhesion protein vinculin at different times after spreading. In *Rac1*^−/−^ fibroblasts, the overall distribution of focal adhesions appeared comparable with that in control cells (Fig. 7A–F). After spreading was complete (24 hours), the major difference between Rac1 control and *Rac1*^−/−^ fibroblasts with regard to their adhesion pattern derived from morphological differences between the two cell types, with *Rac1*^−/−^ fibroblasts being on average less polarized than *Rac1*-expressing control cells. These data clearly revealed that although certainly involved in nascent adhesion assembly (Nobes and Hall, 1995; Rottner et al., 1999a), Rac GTPases are not essential for this process.

We next analyzed spreading by phase-contrast video microscopy, and sought to correlate morphological changes accompanying spreading with early adhesion formation. Time-lapse movies revealed that *Rac1*^−/−^ cells spread rapidly and efficiently by employing protrusive filopodia, which became apparent within 20–60 seconds upon substrate contact (Fig. 7H; supplementary material Movie 5). These filopodia operated as struts for the protrusion of cytoplasm in between. Importantly, the dynamics of spreading of *Rac1*^−/−^ cells was unaffected when overexpressing the C-terminal domain of N-WASP (N-WASP-WWCA) to sequester the Arp2/3 complex in the cytosol (Machesky and Insall, 1998) (supplementary material Fig. S6; Movie 6). Hence, Rac-independent spreading and filopodium formation probably do not involve pathways of Arp2/3 activation as an alternative to the Rac–WAVE signaling axis, consistent with a lack of function of the Arp2/3 complex in filopodium formation (Steffen et al., 2006; Gomez et al., 2007; Nicholson-Dykstra and Higgs, 2008; Suraneni et al., 2012; Wu et al., 2012).

In contrast, spreading of Rac1 control cells was mostly achieved through formation of ruffles and flat lamellipodia (Fig. 7G), as expected from phalloidin and anti-Abi immunolabelings (Fig. 6). Focal adhesion formation shortly after initiation of spreading of *Rac1*^−/−^ cells was also followed by fluorescence video microscopy of the protrusion and adhesion marker VASP (Rottner et al., 1999b). Sequential imaging using phase-contrast and fluorescence microscopy showed strong accumulation of EGFP–VASP in focal adhesions (Fig. 7J; see also supplementary material Movie 7). Phase-contrast movies confirmed the presence of multiple, protrusive filopodia (Fig. 7I). Importantly, VASP failed to target to the peripheral regions of cytoplasm, squeezing forward between filopodial struts, confirming the absence of lamellipodia (Fig. 7J). Interestingly, however, the exploratory, protrusive behavior of filopodia was frequently followed by VASP-associated adhesion formation at their base, reminiscent of the Cdc42-induced focal complexes described previously (Nobes and Hall, 1995) (Fig. 7K; supplementary material Movie 7). Similar adhesions were also observed in CAR fibroblasts rich in filopodia (Nemethova et al., 2008). *Rac1*^−/−^ cells expressing fluorescent paxillin showed essentially the same dynamics of nascent adhesion formation as observed for VASP (supplementary material Fig. S7). Together, our data provide direct evidence that filopodia can mediate cell spreading and seed nascent adhesions equally well as lamellipodia. As a consequence, no delay in spreading or reduction of substratum area covered by these cells was observed. However, this was in strong contrast to the critical function of Rac activity in cytoskeletal reorganizations essential for effective cell translocation and migration.

### Rac deficiency does not affect focal adhesion number and size, but increases mobility of focal adhesion components

**Fig. 8.**
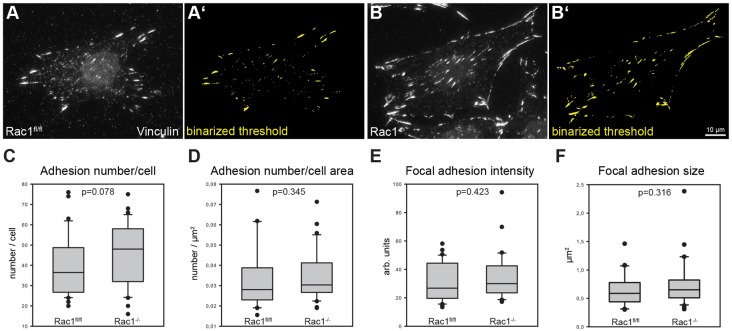
**Analysis of focal adhesion parameters in *Rac1*^−/−^ MEFs.** (A–F) Assessment of focal adhesion parameters from vinculin stainings. Representative images of *Rac1^fl/fl^* (A,A′) and *Rac1*^−/−^ (B,B′) cells stained with vinculin antibodies (A,B). Images were thresholded as detailed in Materials and Methods; a binarized threshold (A′,B′) is shown to represent data extraction. From these images, number of adhesions per cell (C), number of adhesions per cell area (D), focal adhesion intensity (E) and focal adhesion size (F) were calculated. 30 cells were analyzed for each cell type.

Next, we assessed quantitative parameters of focal adhesion patterns as well as turnover of different focal adhesion components. Using acute Cre-recombinase-mediated Rac1 gene deletion in primary MEFs, Guo and colleagues concluded that Rac1 is crucial for both focal adhesion and stress fiber assembly (Guo et al., 2006). Focal adhesions stained with vinculin antibodies were identified using image thresholding (Fig. 8A,A′,B,B′). Interestingly, *Rac1*^−/−^ cells displayed increased adhesion numbers per cell (Fig. 8C), but this difference was mostly due to increased cell areas in the absence of Rac (Fig. 6; Fig. 8D). In addition, average focal adhesion intensity (Fig. 8E) and size (Fig. 8F) were roughly equal in both cell types. Although total intensity of vinculin staining per cell was increased upon Rac deletion in a statistically significant fashion (supplementary material Fig. S8), this increase appeared insignificant when expressing vinculin intensity relative to cell area. Moreover, the modest increase of adhesion area per cell with or without normalization to increased cell area in *Rac1*^−/−^ cells was also statistically insignificant (supplementary material Fig. S8C,D).

**Fig. 9.**
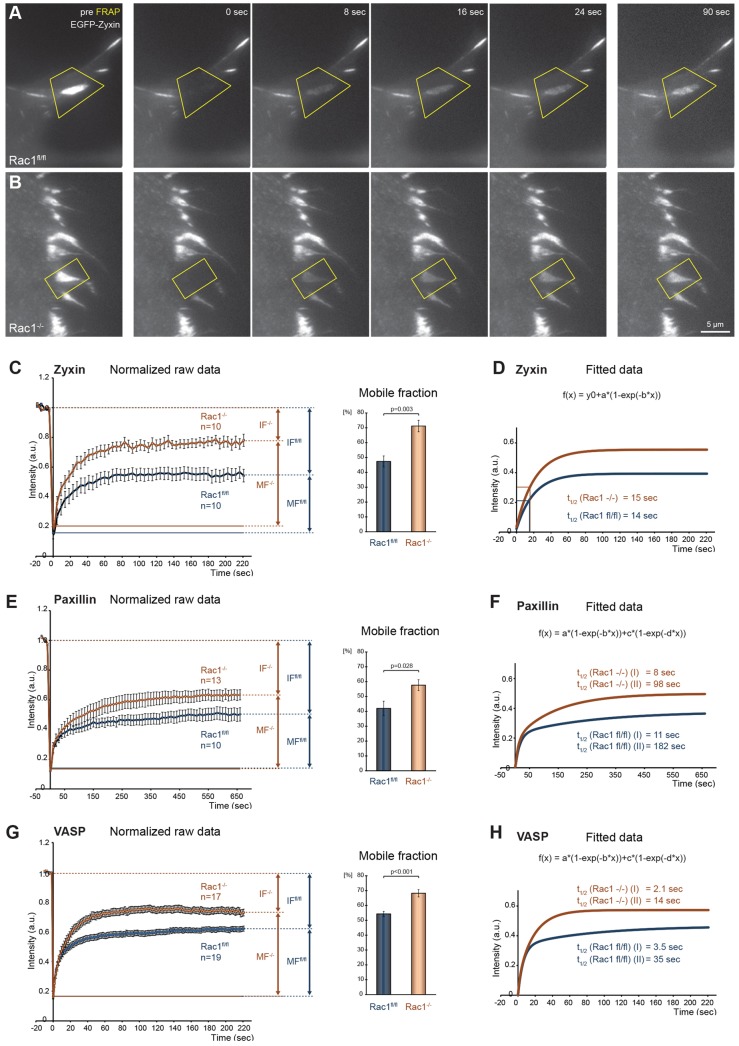
**Turnover of focal adhesion components.** (A–D) FRAP analysis of EGFP–zyxin in *Rac1^fl/fl^* (A) and *Rac1*^−/−^ (B) MEFs. Representative frames of bleaching experiments show EGFP–zyxin accumulation in focal adhesions before (pre FRAP, left panel), immediately after bleaching (0 seconds) and during fluorescence recovery at the time points indicated. Yellow quadrilaterals mark bleached areas. (C–H) Recovery curves of normalized fluorescence intensities of EGFP-tagged zyxin (C,D), paxillin (E,F) and VASP (G,H) in *Rac1^fl/fl^* (blue) and *Rac1*^−/−^ cells (orange). C,E,G show arithmetic means with s.e.m. for acquired time points before and after bleaching. MF, mobile fraction, IF, immobile fraction. Mobile fractions are shown as percentages of the total fraction (sum of mobile and immobile fraction). D,F,H show fitted curves of averaged data from which half times of recovery (*t*_1/2_) were calculated. *N* = number of analyzed movies; respective equations of curve fits for each component are displayed in the figure, equation coefficients are given in supplementary material Table S3.

Finally, we tested whether Rac deficiency had consequences on protein turnover in individual focal adhesions. EGFP-tagged zyxin readily incorporated upon transient expression in both cell types (Fig. 9A,B; supplementary material Movie 8), as expected (Rottner et al., 2001). Zyxin is known to turn over within focal adhesions within seconds (Lele et al., 2006; Wolfenson et al., 2011). Interestingly, although the turnover of zyxin was very similar in both the presence and absence of Rac1 (*t*_1/2_ of roughly 14 and 15 seconds, respectively), the mobile fraction of zyxin in focal adhesions was substantially increased in the absence of Rac (Fig. 9C,D). Interestingly, a similar increase was found for distinct focal adhesion components, paxillin (Fig. 9E,F; supplementary material Fig. S9A) and VASP (Fig. 9G,H; supplementary material Fig. S9B). In addition, the recovery behavior of both paxillin and VASP was more complex in both cell types, compared with that of zyxin. Curve fits suggested two differentially exchanging fractions for paxillin and VASP, and revealed an increase in turnover for the slowly exchanging fraction. However, common to all components examined, fluorescence recovery after photobleaching (FRAP) data revealed a shift towards greater immobile fractions in the presence of Rac. Collectively, these data suggest that although being dispensable for adhesion site initiation, stabilization and adhesion component turnover (Figs 7–9; supplementary material Figs. S7–S9), the focal complex assembly phenotype induced upon microinjection of active GTPase (Nobes and Hall, 1995; Rottner et al., 1999a) may derive from Rac promoting association of focal adhesion constituents within these structures or their stabilization.

## Discussion

In order to understand Rac1 functions in fibroblast morphology, migration and dynamic actin rearrangements, we developed Rac1-deficient fibroblastoid cells. Importantly, the generation of individual and viable Rac1 knockout clones made possible, for the first time, a detailed molecular analysis of the requirement of Rac1 function for protrusion, adhesion, random and directed migration and spreading. Of note, bearing in mind previous attempts to eliminate the Rac1 gene in fibroblasts, Rac1 was concluded to be essential for proliferation (Guo et al., 2006; Vidali et al., 2006). In our permanent knockout cell lines, we can exclude the presence of residual Rac1 protein in our cell populations, and were unable to detect Rac2 and Rac3 at the protein level. As expected, our results revealed Rac1 to be central to spontaneous lamellipodium formation and growth-factor-mediated dorsal ruffling in fibroblasts, corroborating previous observations (Guo et al., 2006; Vidali et al., 2006). These results are most likely explained by the key role played by Rac in relaying signals to the actin polymerization machinery through the WAVE complex (Kunda et al., 2003; Rogers et al., 2003; Innocenti et al., 2004; Steffen et al., 2004). We also examined regulation of lamellipodium formation by other Rho GTPases. We could show that although Rac2 and Rac3 were as efficient as Rac1 in inducing lamellipodium formation, neither RhoG nor Cdc42 were able to drive lamellipodia independently of Rac1. Consistently, all Rac variants were observed to interact with the WAVE complex, unlike RhoG and Cdc42. From recent experiments, a potential function of RhoG in driving Rac-independent membrane ruffling and migration was proposed (Meller et al., 2008). However, since clonal *Rac1*-null cells could not be employed in this study, cell populations might have been contaminated with un-recombined *Rac1* alleles (Vidali et al., 2006). In addition, signaling to the actin polymerization machinery remained elusive, as no GTP-loading-specific interaction of RhoG with WAVE complex could be observed (Meller et al., 2008), as confirmed in our experiments. Based on the results presented here using permanent cell lines lacking detectable Rac activity, we speculate that RhoG can only induce ruffling in the presence of residual Rac activity, and is thus incapable of bypassing Rac signaling to actin remodeling. We can also exclude that Cdc42 is able to drive lamellipodium formation in the absence of Rac, consistent with the specificity of interaction of each GTPase with Arp2/3 complex activators. Cdc42-GTP activates the Arp2/3 complex through N-WASP (Rohatgi et al., 1999), which is involved for instance in vesicle trafficking and endocytosis, but not in lamellipodium or filopodium formation (Lommel et al., 2001; Snapper et al., 2001; Benesch et al., 2002; Czuchra et al., 2005). In contrast, Rac-GTP triggers Arp2/3-complex-dependent actin remodeling through the WAVE complex (Ismail et al., 2009; Lebensohn and Kirschner, 2009).

Our cell lines should also prove instrumental in dissecting more carefully the requirements of post-translational modifications of GTPases in conjunction with subcellular positioning and activation. For instance, prenylation and postprenylation processing of the C-terminus of Rac1 have long been considered essential for plasma membrane targeting and hence actin remodeling events. The CLLL sequence at the C-terminus of Rac1 is first geranylgeranylated by GGTase-I, followed by removal of AAX by the prenyl-CAAX-specific protease Rce1 (Ras converting enzyme 1) (Boyartchuk et al., 1997), and by carboxyl methylation of the isoprenylcysteine by Icmt (isoprenylcysteine-directed carboxyl methyltransferase) (Dai et al., 1998). In spite of the crucial functions ascribed to these and the associated post-translational modifications of Rho GTPases (Navarro-Lérida et al., 2012), some recent studies with genetic deletion of these different enzymes have challenged this view. For instance, removal of GGTase-I in macrophages did not decrease levels of active GTPases, but strongly increased them (Khan et al., 2011). Moreover, fibroblasts with genetic deletions of Rce1 or Icmt exhibited minor effects on Rac-induced actin remodeling (Michaelson et al., 2005), questioning the proposed requirement of CAAX-processing for proper Rho-GTPase function. Our experiments can shed light on this controversy, as expression of a constitutively active Rac1-ΔCAAX in *Rac1*^−/−^ cells reconstituted lamellipodia, albeit less efficiently as constitutively active full length Rac1. Since Rac1-ΔCAAX was strongly reduced in the membrane fraction, it is tempting to speculate that Rac1 plasma membrane association might be less relevant for correct WAVE complex positioning than previously thought. Future work will have to clarify how processing and activation of Rac and WAVE complex are spatially and temporally correlated.

Rac1 deletion completely abolished ruffle and lamellipodium formation and strongly reduced migration capacity, arguing for the requirement of lamellipodia for efficient cell motility. Our cells showed a more dramatic migration phenotype than observed previously (Vidali et al., 2006). Again, the efficiency of Rac1 depletion, in the latter case reported to be ∼90%, is reasonably consistent with mild migration defects observed in RNAi studies (Monypenny et al., 2009). We speculate that residual Rac1 protein activities present in these earlier experimental systems were sufficient to mediate reasonably effective migratory performance. Nevertheless, our permanent Rac1-deficient cells also displayed residual migration, which appeared mostly accompanied by protrusion of filopodia and cytoplasm between them, together with formation of contractile bundles, seemingly employed for translocation of the cell body. Surprisingly, however, inhibition of RhoA signaling to contraction increased instead of decreased both Rac-dependent and -independent migration. At first glance, this might be counterintuitive, since Rho and Rac signaling were previously reported to be mutually antagonistic to different modes of migration (Sanz-Moreno et al., 2008). However, our data indicate that contractility as induced on two-dimensional surfaces mostly counteracts migration, as observed previously for RhoA stimulation (Nobes and Hall, 1999), irrespective of Rac signaling. We conclude that efficient, mesenchymal migration on two-dimensional surfaces, as studied here, requires Rac signaling. In addition, this type of migration is clearly suppressed by exaggerated myosin-II-based contractility in a fashion separable from Rac signaling. In future work, it will be exciting to assay migration behavior and speed of *Rac1^−/−^* fibroblasts in three-dimensional matrices.

An ineffective mode of migration involving filopodia as observed in Rac-deficient cell lines was recently also observed in fibroblasts stably suppressed by Arp2/3 complex RNAi (Wu et al., 2012). In these cells, migration rates were reduced only to 50% of control cells. So whether the more penetrant phenotype of Rac-deficient cells derives from more efficient inhibition of the Rac/WAVE/Arp2/3 complex signaling axis or from additional, WAVE- and Arp2/3-independent functions of Rac remains to be investigated. Whatever the case, removal of Rac expression coincided with lack of continuous accumulation of WAVE complex at the cell periphery, and thus probably a lack of Arp2/3 complex activation at these sites. Whether or not the lack of Arp2/3 activation at the cell periphery of Rac-deficient cells is causative of the reduced migration rates observed remains to be tested, especially in light of the lack of phenotype in random cell migration observed recently in ArpC3-deficient cells (Suraneni et al., 2012).

Irrespective of strong effects on random and directed migration, Rac deficiency caused an additional, separable defect in the ability of cells to sense and follow a chemotactic signal. Future experiments will explore whether this defect arises from problems with signal transduction or propagation or with establishment and maintenance of polarity.

Equally surprising, considering the absence of lamellipodia and severe reduction of migration upon Rac removal, was the capability of these cells to spread efficiently and to form focal adhesions. However, careful analysis of morphological changes after cell seeding revealed that spreading is mediated by prominent filopodia, the substratum-attached shafts of which appeared to seed the formation of nascent adhesions. Although these data disagree with one previous study (Guo et al., 2006), for reasons that remain to be determined, they unequivocally establish that Rac activity is not essential for both filopodia and adhesion formation as well as cell spreading. Although numbers and sizes of focal adhesions were virtually unaffected in the absence of Rac, assessment of turnover of individual focal adhesion components revealed a modest but clearly detectable phenotype. For all components tested, FRAP experiments revealed Rac deficiency to cause a marked increase in adhesion component mobility. This suggests that Rac contributes to nascent adhesion formation through adhesion component stabilization.

In summary, using gene disruption methodology in differentiated fibroblastoid cells, we present here the first permanent and viable Rac1-deficient cell system. Our data underline the importance of Rac signaling for efficient cell migration. Moreover, induction of lamellipodia by RhoG and Cdc42 strictly depends on Rac GTPases. The most striking feature of the peripheral actin cytoskeleton of Rac1-deficient cells is the formation of multiple, protrusive filopodia. Although these structures fail to contribute effectively to random or directed migration, they are sufficient to mediate cell spreading. Apart from migration, Rac has additional, yet undefined functions in chemotactic signaling, and might contribute to stabilization of focal adhesions components. Our permanent, Rac-deficient cell lines will be helpful for dissecting the functions of this relevant subfamily of Rho GTPases in diverse cellular processes.

## Materials and Methods

### Generation of *Rac1^fl/fl^* and *Rac1*^−/−^ MEFs

*Rac1^fl/fl^* fibroblastoid cells were generated by immortalization of primary mouse embryonic fibroblasts prepared from embryonic day (E)14.5 *Rac1^fl/fl^* embryos with SV40 large T transducing retrovirus. To generate clones homozygously deleted for the Rac1 gene, *Rac1^fl/fl^* MEFs were transiently transfected with pCre-Pac (Taniguchi et al., 1998) and selected with 5 µg/ml puromycin for 2 weeks. Individually growing cell clones were isolated and analyzed by genotyping and western blotting.

### Genotyping

Genomic DNA was isolated from MEFs as described previously (DeChiara, 2001). Genotyping for the floxed (fl) and *Rac1*^−/−^ (knockout PCR) alleles was performed as described previously (Chrostek et al., 2006).

### Cell culture and transfection

MEFs were maintained in DMEM, 4.5 g/l glucose supplemented with 10% FCS (Sigma), 2 mM L-glutamine, 0.1 mM non-essential amino acids and 1 mM sodium pyruvate. MEFs were transfected with FuGENE 6 (Roche) or JetPei (Polyplus). For analysis of Rac1-N17, pRK5-myc-Rac1-N17 was co-transfected with a plasmid mediating strong GFP expression (psiRNA-h7SKGFPzeo) in a ratio of 2∶1. B16-F1 cells were maintained and transfected as described previously (Steffen et al., 2004). The plasmids used in this study are listed in supplementary material Table S1.

### Recombinant proteins

All recombinant proteins were overexpressed in *E. coli* BL21(DE3)pLysS (GE Healthcare) according to standard procedures, using glutathione–Sepharose (GE Healthcare) and amylose resin (NEB). Proteins bound to the matrix were snap frozen in 50 mM Tris-HCl, 150 mM NaCl, 5 mM MgCl_2_, 1 mM DTT, protease inhibitors (Mini Complete, EDTA free, Roche), pH 7.5 supplemented with 10% glycerol. Proteins for microinjection were purified as described previously (Ridley et al., 1992).

### Pull down

Recombinant proteins (200 pmol) bound to resin were incubated with 800 µg cleared B16-F1 cell lysates in lysis buffer L1 [50 mM Tris-HCl, 50 mM NaCl, 15 mM KCl, 15 mM MgCl_2_, 1 mM EGTA, 20 mM NaF, 1% (w/v) polyethylene glycol 3350, 1% Triton X-100, pH 7.5 and protease inhibitors (Mini Complete, EDTA free, Roche)] for 1 hour at 4°C. The matrix was washed three times in buffer L1 without NaF and Triton X-100. Bound proteins were analyzed by western blotting according to standard procedures. Antibodies, reagents and treatments are listed in supplementary material Table S2.

### Membrane fractionation

Cells were trypsinized, washed three times and homogenized by 3–10 strokes through a 27 gauge needle in 10 mM Tris, pH 7.5, 1 mM EDTA, 150 mM NaCl, 1 mM DTT and protease inhibitors (Mini Complete, EDTA free, Roche) until 50–70% of the cell were broken. Intact cells and nuclei were separated by centrifugation at 500 ***g*** at 4°C for 5 minutes. Supernatants were subjected to ultracentrifugation at 100,000 ***g*** at 4°C for 30 minutes. Cytosolic fractions were removed and pelleted membranes were washed by overlaying once with buffer. Samples were analyzed by western blotting.

### Immunofluorescence

For immunofluorescence analyses, MEFs were seeded onto acid (HCl)-washed coverslips prepared as follows. For cells shown in Fig. 4E–J, coverslips were pre-incubated with 500 µl growth medium per well of a 24-well plate overnight. In all other cases, coating was carried out by preparing a dilution of the respective matrix and placing a 100 µl drop onto a 12 mm coverslip. Fibronectin (FN; #11051407001, Roche) was dissolved at 1 mg/ml in 2 M urea. For coatings, FN was diluted in PBS to 25 µg/ml except for the spreading assays shown in Fig. 6G, where FN was used at 5 µg/ml. FN was coated for 1 hour at room temperature or overnight at 4°C. Coverslips were then washed three times with PBS and overlayed with growth medium. Gelatin (G1393, Sigma) was coated at 0.2% in PBS for 1 hour, washed once with PBS and replaced with growth medium. Laminin (L-2020, Sigma) was diluted to 25 µg/ml in 50 mM Tris, pH 7.4, 150 mM NaCl and coated for 50 minutes, washed once with PBS and replaced with growth medium. For all fixations, solutions were pre-warmed to 37°C. For vinculin stainings, MEFs were washed with cytoskeleton buffer (CB) (Rottner et al., 1999b), pre-extracted with 0.3% Triton X-100 in CB for 1 minute and fixed with 4% paraformaldehyde (PFA) in CB for 20 minutes. For all other stainings, MEFs were washed with CB, fixed with 4% PFA in PBS or CB for 20 minutes and permeabilized with 0.1% Triton X-100 in PBS or CB for 60 seconds. Cells were blocked with 5% horse serum in 1% BSA in PBS or CB and then stained with the indicated antibodies and/or phalloidin.

### Image acquisition

Images were captured using 40×/1.3 NA Plan-Neofluar and 63×/1.4 NA Plan-Apochromat oil objectives on an inverted Zeiss Axiovert 100TV equipped with an HXP 120 lamp (Visitron) for epifluorescence illumination, a halogen lamp for phase-contrast imaging, a Coolsnap-HQ2 camera (Photometrics) and shutter drivers (Uniblitz Corporate) driven by Metamorph software (Molecular Devices). Alternatively, an inverted Axio Observer (Zeiss, see also below for random migration and FRAP experiments) equipped with an automated stage, a DG4 light source (Sutter Instrument) for epifluorescence illumination, a VIS-LED for phase-contrast imaging, and a Coolsnap-HQ2 camera (Photometrics) driven by VisiView software (Visitron Systems) was used.

### Time-lapse video microscopy, microinjection and FRAP

Live cell imaging and microinjection was performed as described previously (Rottner et al., 1999a; Rottner et al., 2001). Spreading of cells was monitored by immediate acquisition of movies after pipetting cell suspensions onto fibronectin-coated coverslips equilibrated with microscopy medium. Wound healing assays of cells seeded into 12- or 6-well plates were carried out as described previously (Lai et al., 2009). For random migration assays, cells were seeded subconfluently in a 6-well plate. After 6 hours, the plate was mounted on an inverted Axio observer (see above) equipped with a 37°C incubator and CO_2_-aerated lid. Phase-contrast movies were acquired on different randomly chosen positions. For Rac1-N17 experiments, expressing cells were identified in the green channel. For chemotaxis assays, μ-Slide Chemotaxis^2D^ chambers (Ibidi) were employed according to manufacturer's instructions with adapted cell concentrations. *Rac1^fl/fl^* and *Rac1*^−/−^ MEFs were adjusted to 1.5×10^6^ and 1×10^6^ cells/ml, respectively. The upper chamber was filled with 2.5% FCS and 100 ng/ml HGF in DMEM (C_100_) as chemoattractant. FRAP experiments were performed on an inverted Axio Observer (Zeiss, see above) using a 63×/1.4NA Plan-Apochromat oil immersion objective. EGFP–zyxin, –paxillin and –VASP were rapidly bleached in selected regions employing the 2D-VisiFRAP Realtime Scanner (Visitron Systems) using 65–90 mW output power of a 405 nm diode laser (Visitron Systems) to achieve nearly complete bleaching for each focal adhesion.

### Data analysis and processing

ImageJ and MetaMorph were used to adjust brightness and contrast levels. For the analysis of spreading areas, cells were stained with phalloidin and randomly chosen fields were acquired using a 40×/1.3 NA objective. Cell area was assessed by manual adjustment of the threshold function for each individual cell (ImageJ). For wound healing, the average distance of wound closure per time point was determined as follows. The area not covered by cells was outlined in ImageJ using the freehand tool for individual frames. Measured areas were divided by the width of chip size to obtain the wound distance. Average wound distances were then plotted over time. Wound closing speed was calculated by dividing the wound distance by the time point of wound closure. For Rac1 knockouts, wound distance was divided by the maximal recording time (20 hours). For random migration and chemotaxis analyses, the manual tracking and chemotaxis tool plugins of ImageJ were used. Cells were tracked throughout all frames or until they disappeared from the viewing area, entered mitosis or collided with other cells.

For focal adhesion quantification, the maximum intensity of all images was adjusted to 900, cells were outlined and threshold adjusted manually to avoid artificial over or under filling of areas. Average intensity per cell, cell area and thresholded area per cell were computed. The number of focal adhesions per cell was counted manually, since thresholded objects were occasionally not separable and tiny spots erroneously counted as focal adhesions.

For FRAP analysis, intensity values of the background outside of the cell were subtracted from the intensity of bleached focal adhesion (‘focal adhesion FRAP’) and background intensity outside of the cell was subtracted from two averaged non-bleached regions inside the cell (‘acquisition photobleaching’). Focal adhesion FRAP was divided by acquisition photobleaching and normalized to the mean intensity values of frames before photobleaching. These data were averaged and plotted as ‘raw data’ using Microsoft Excel. To estimate mobile fractions for each individual experiment, the last five curve values were averaged, followed by subtraction of fluorescence after photobleaching, as indicated in Fig. 9C,E,G, and expressed as a percentage of the total fraction. For data fitting, average curves were normalized to *y* = 0 for the first time point after bleaching. Data were fitted in SigmaPlot 12.0 (Systat Software) using dynamic curve fits for exponential rise to maximum. Zyxin data best fitted a mono-exponential function, whereas paxillin and VASP fitted bi-exponential functions, as shown in Fig. 9. Curve values obtained from SigmaPlot were calculated for each time point using Excel, and plotted as ‘fitted data’. In the case of zyxin, half times of recovery were calculated using equation 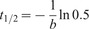. In the case of paxillin and VASP, half times of recovery for rapidly and slowly exchanging fractions were calculated using equations 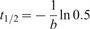 and 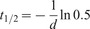, respectively.

Statistical analysis was performed using the non-parametric Mann–Whitney rank sum test (SigmaPlot 12.0). Data were processed using Microsoft Excel 14, ImageJ 1.43u (http://rsbweb.nih.gov/ij/), SigmaPlot 12.0 (Systat Software), Adobe Photoshop 11.0.2 and Adobe Illustrator 14.0.0.

## Supplementary Material

Supplementary Material
