# Supplementary material for: Rac function is crucial for cell migration but is not required for spreading and focal adhesion formation
Source: J Cell Sci. 2013 Oct 15;126(20):4572–88. doi: 10.1242/jcs.118232 (PMC3817791; doi:10.1242/jcs.118232)
Supplement: Supplementary Material [file supp_126_20_4572__index.html]

Rac function is crucial for cell migration but is not required for spreading and focal adhesion formation — Supplementary Material 

# Rac function is crucial for cell migration but is not required for spreading and focal adhesion formation

## JCS118232 Supplementary Material

**Files in this Data Supplement:**

- **Supplementary Material PDF**
