# Supplementary material for: Rac function is crucial for cell migration but is not required for spreading and focal adhesion formation
Source: J Cell Sci. 2013 Oct 15;126(20):4572–88. doi: 10.1242/jcs.118232 (PMC3817791; doi:10.1242/jcs.118232)
Supplement: Supplementary Material [file supp_126.20.4572_JCS118232.pdf]

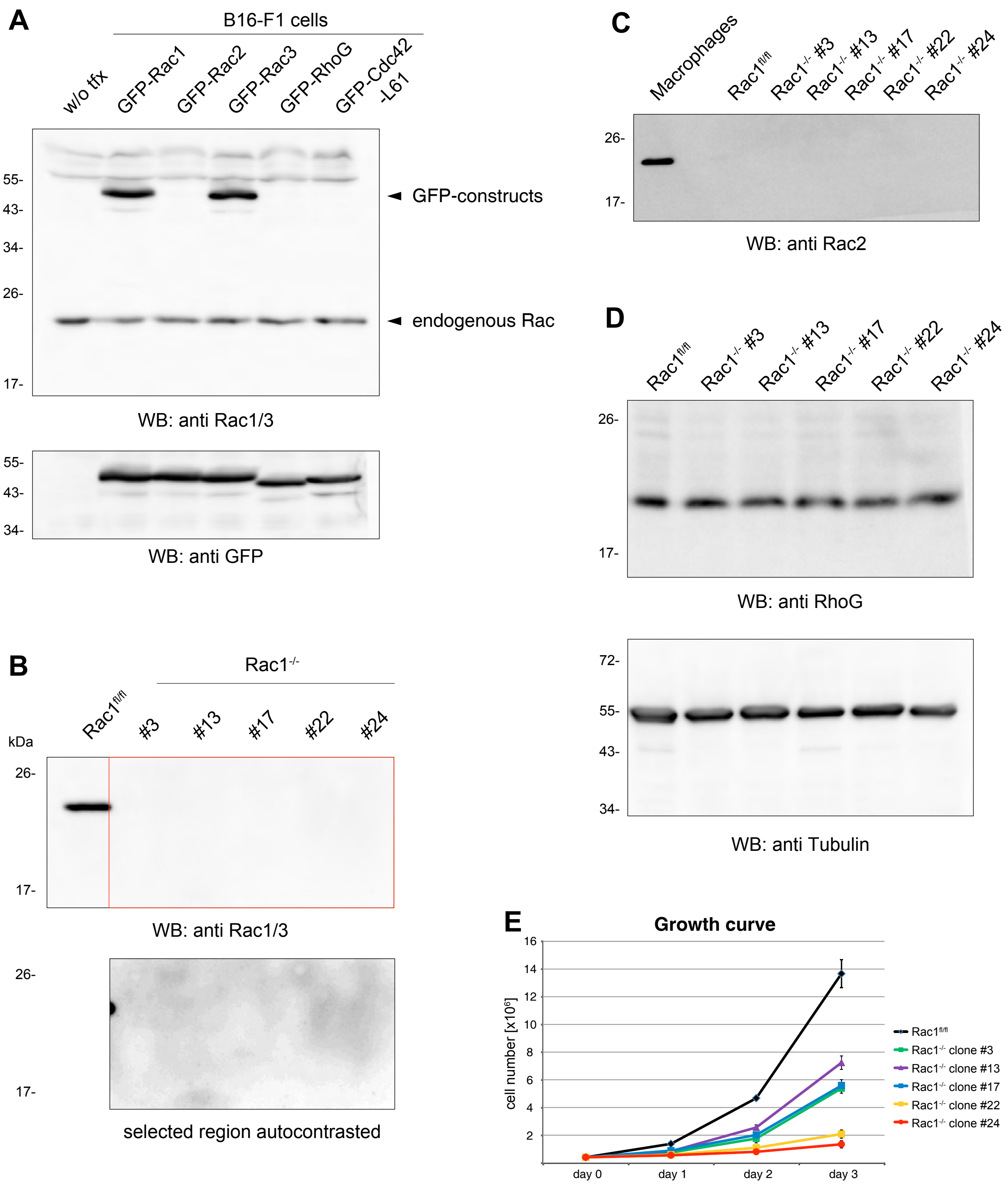

Fig. S1. Rac1$^{-/-}$ MEFs lack Rac2 and Rac3 and display unchanged RhoG levels. (**A**) The antibody used in Figure 1B recognises Rac1 and Rac3. Western blot of untransfected B16-F1 cells (w/o tfx) and B16-F1 cells overexpressing GFP-fusion constructs as indicated was developed with Rac1/3 antibody (upper panel) employed in Figure 1B and Supplementary Figure S1B. Western blot of anti-GFP (lower panel) was used to show equal expression levels of GFP-constructs. (**B**) A region from the Western Blot shown in Figure 1B (red rectangle in the upper panel) was autocontrasted (lower panel). Note that Rac1$^{-/-}$ MEFs do not express any detectable levels of Rac1 and Rac3. (**C**) Rac2 is not expressed in Rac1$^{-/-}$ clones. Western blot of macrophages, Rac1$^{fl/fl}$ and Rac1$^{-/-}$ clones as indicated was developed with Rac2-specific antibody. (**D**) RhoG protein levels are unaffected in Rac1$^{-/-}$ MEFs. Western blot of Rac1$^{fl/fl}$ and individual Rac1$^{-/-}$ clones as indicated was developed with RhoG antibody (upper panel). Anti-Tubulin was used as loading control (lower panel). (**E**) Growth curves of Rac1$^{fl/fl}$ and five individual Rac1$^{-/-}$ clones (clone #3, #13, #17, #22, #24). Cells were seeded at 4 x 10^5 cells per 10 cm dish and total cell numbers determined on the following three days. Data points correspond to arithmetic means and s.e.m. from three independent experiments.

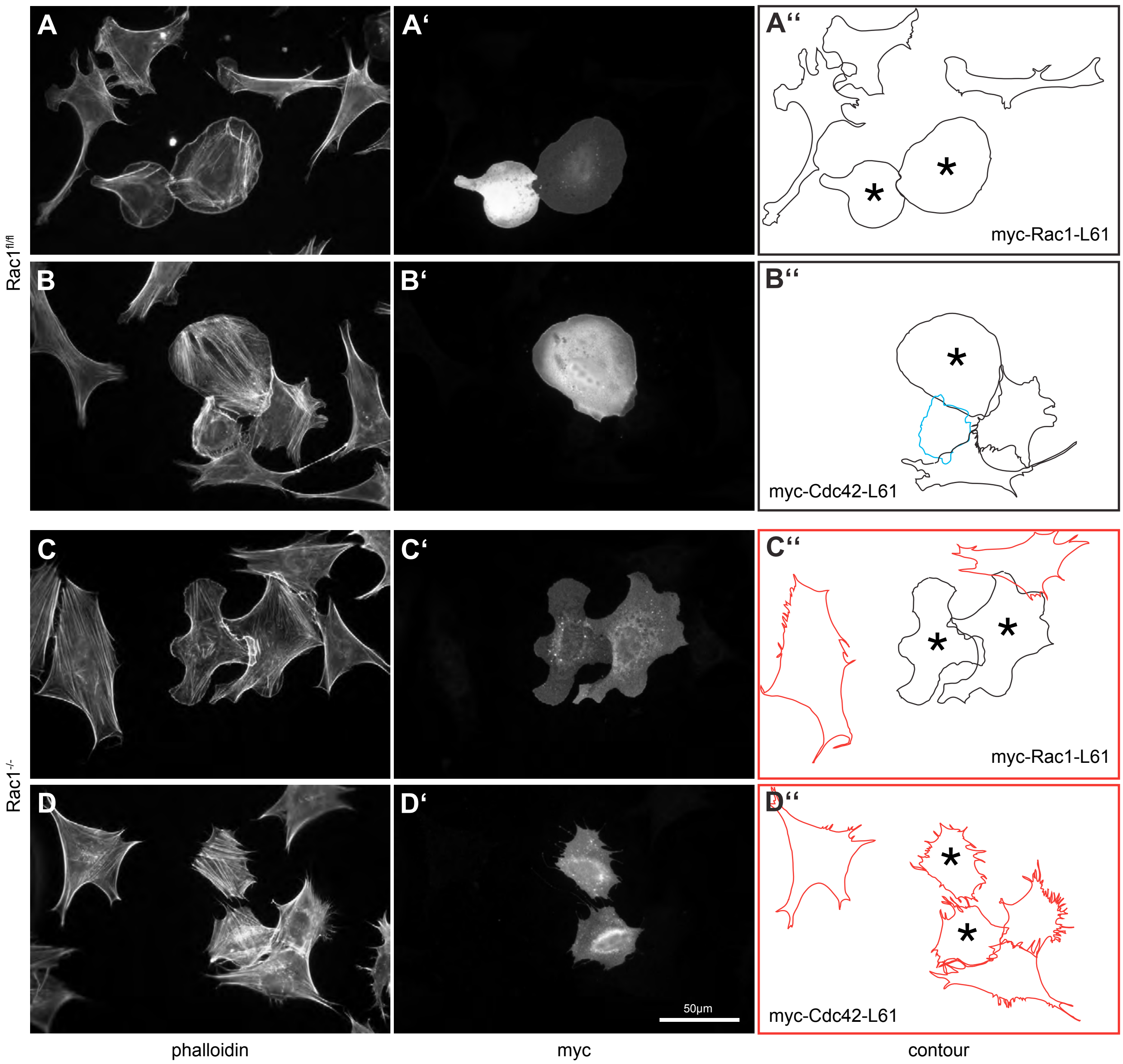

Fig. S2. Overview of cell morphologies with and without different Rho- GTPase expression. Rac1$^{fl/fl}$ (**A**, **B**) and Rac1$^{-/-}$ cells (**C**, **D**) were transfected with myc-Rac1-L61 (**A**, **C**) and myc-Cdc42-L61 (**B**, **D**), fixed and stained with phalloidin (A, B, C, D) to visualize the actin cytoskeleton and anti-myc (**A'**, **B'**, **C'**, **D'**) to identify transfected cells. All cells were outlined and Rho-GTPase expressers marked with an asterisk (**A''**, **B''**, **C''**, **D''**). Black outline colour indicate cells that were categorized as harbouring "lamellipodia", red outlines indicate cells that were categorized as harbouring "filopodia", and blue outline indicates a cell that was categorized as "without protrusion" (see also Figure 2G).

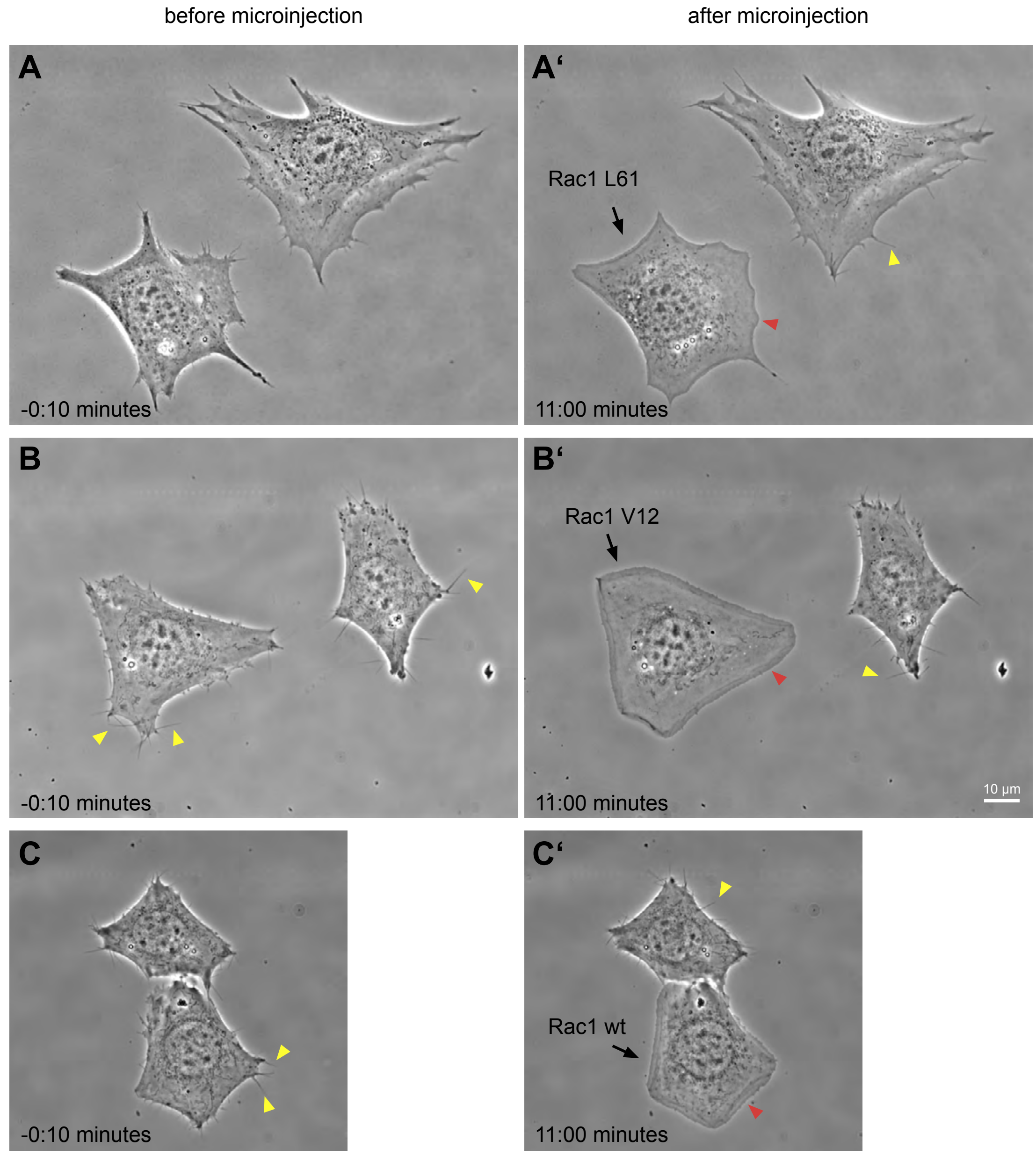

Fig. S3. Microinjection of purified Rac1 proteins into Rac1$^{-/-}$ cells induces prompt lamellipodia formation. Phase contrast images of Rac1$^{-/-}$ fibroblasts 10 seconds before (**A**, **B**, **C**) and 11 minutes after microinjection of Rac1 proteins as indicated (**A'**, **B'**, **C'**). The injected cell is marked with the black arrow in each case. Constitutively active Rac1 mutants (Rac1-L61, A', Rac1-V12, B') as well as wild type Rac1 induce lamellipodia formation (red arrowheads). Note that spontaneous, protrusive filopodia (yellow arrowheads) completely disappear upon microinjection of all Rac1 variants.

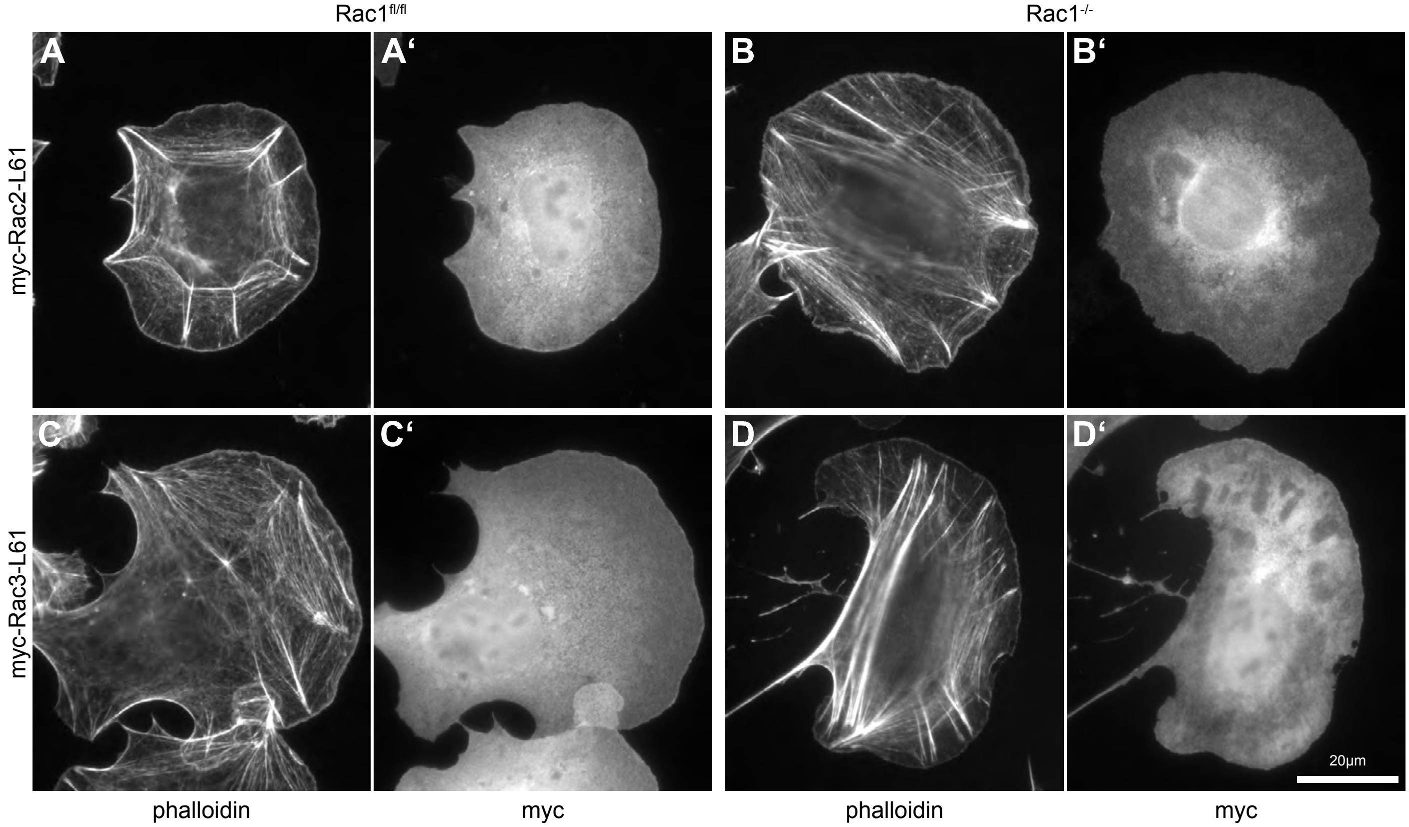

Fig. S4. Rac2 and Rac3 can restore lamellipodia formation in Rac1$^{-/-}$ MEFs. (**A-D'**) Rac1$^{fl/fl}$ (A, A', C, C') and Rac1$^{-/-}$ (B, B', D, D') MEFs were transfected with myc-tagged Rac2-L61 (A-B') and Rac3-L61 (C-D'), fixed and stained with phalloidin (A, B, C, D) and anti-myc (A', B', C', D'). Note the presence of lamellipodia in control and Rac1$^{-/-}$ cells.

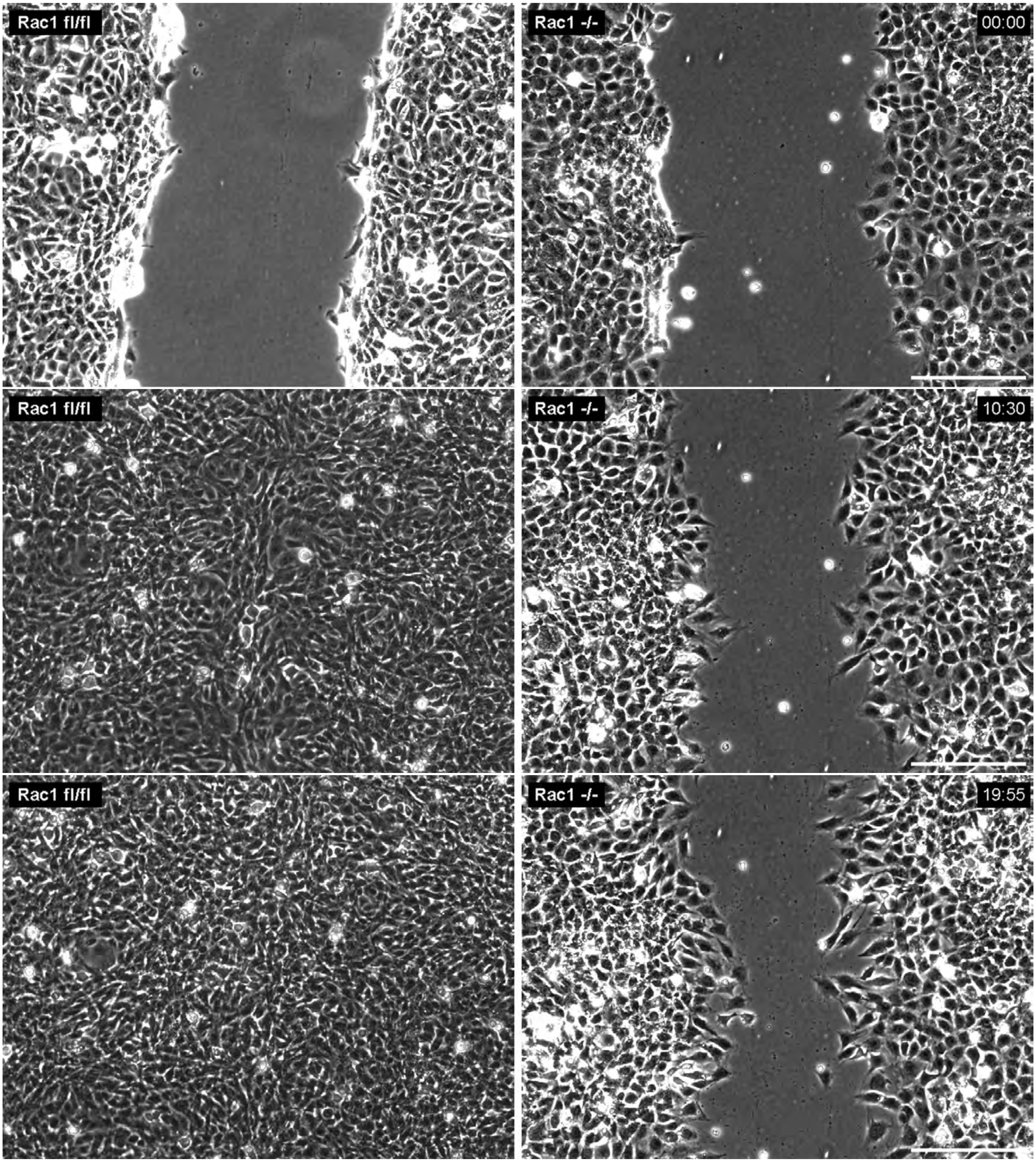

Fig. S5. Rac1$^{-/-}$ cells are unable to close the wound within 20 hours. Related to Fig. 3A. Selected frames from wound healing movies of Rac1$^{fl/fl}$ (left panel) and Rac1$^{-/-}$ cells (right panel). Time is given in hours and minutes. Scale bars equal 200 μm. Note that Rac1$^{-/-}$ cells are unable to close the wound even after almost 20 hours.

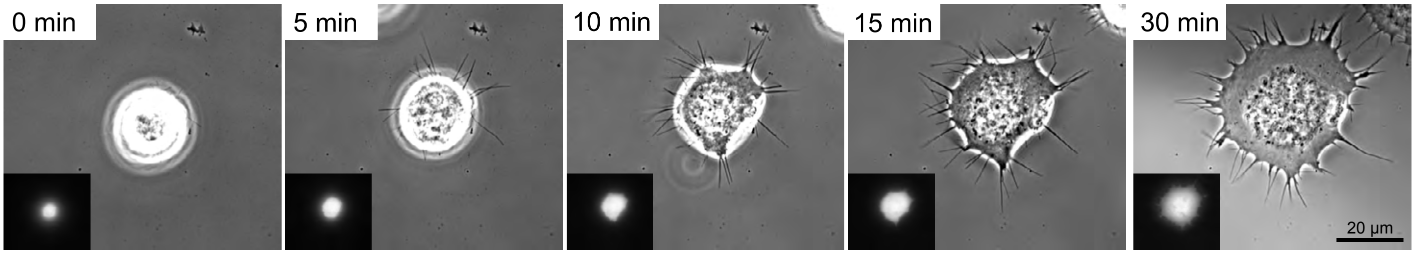

Fig. S6. Rac1$^{-/-}$ MEFs expressing GFP-N-WASP-WWCA are also capable of potent cell spreading. Selected frames of representative phase contrast movie of Rac1$^{-/-}$ cell acquired during spreading. The inset shows acquisition of the green fluorescent channel at respective time points.

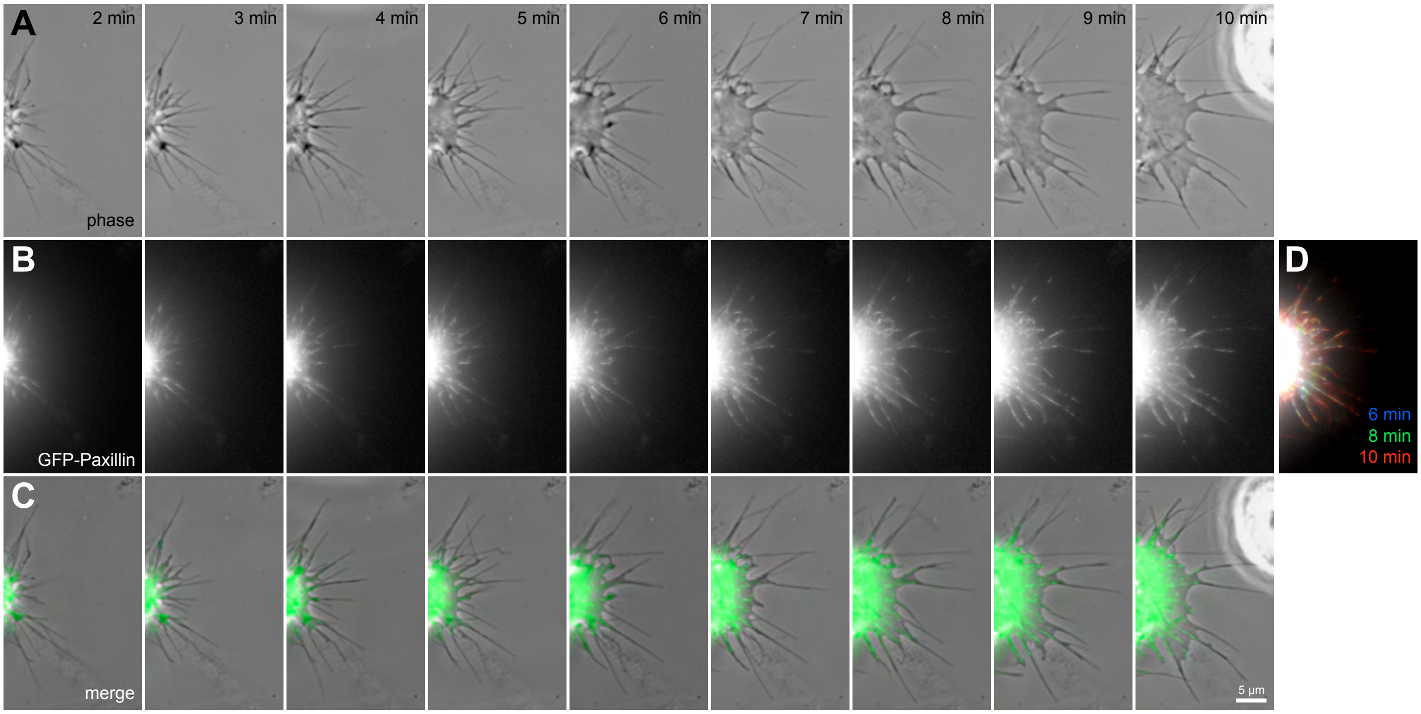

Fig. S7: Rac1$^{-/-}$ MEFs form Paxillin-containing adhesions at the base of filopodia during spreading. Selected frames of phase contrast (**A**) and green epifluorescence channel (**B**) of Rac1$^{-/-}$ cells expressing GFP-Paxillin show accumulation of this focal adhesion protein at the base of protruding filopodia. (**C**) Merged phase contrast (grey) and GFP-Paxillin (green) frames. Time gives minutes after substrate contact. (**D**) Maturation of focal adhesions over time is shown in a merge of GFP-Paxillin images at different time points. Blue corresponds to the GFP-Paxillin pattern after 6 minutes of spreading, green after 8 and red after 10 minutes.

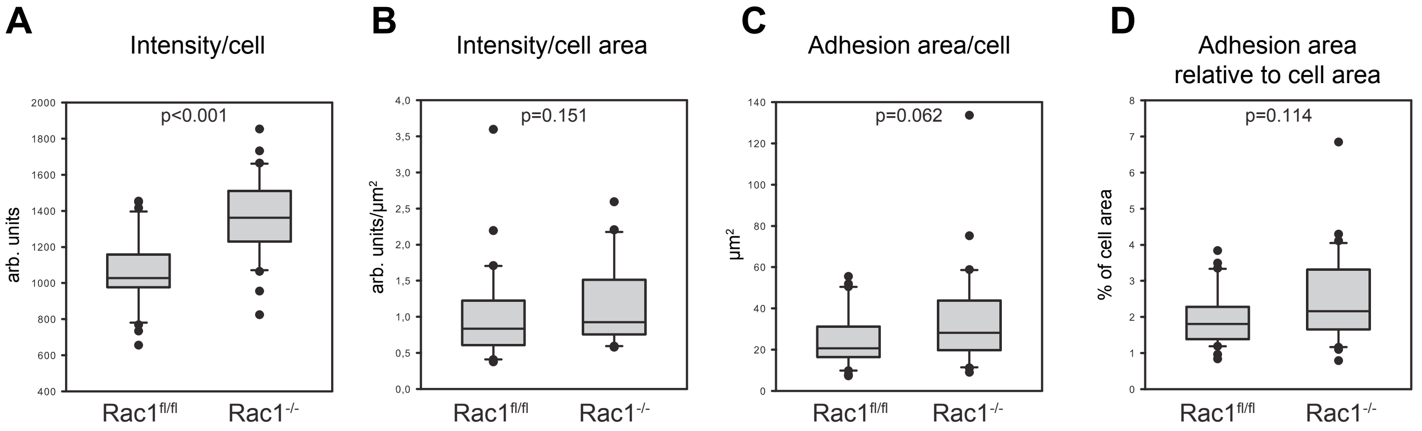

Fig. S8. Additional focal adhesion parameters in Rac1$^{-/-}$ MEFs. Vinculin stainings of Rac1$^{fl/fl}$ and $^{-/-}$ MEFs (see Figure 8A, B) were processed as described in material and methods. Graphs show (**A**) adhesion intensity per cell (medians 1028 and 1362, respectively), (**B**) adhesion intensity per cell area (medians 0.834 and 0.924, respectively) (**C**) adhesion area per cell (medians 20.7 and 28.2, respectively) and (**D**) adhesion area relative to the cell area (medians 1.81 and 2.16, respectively). Box and whiskers plots show medians, 10th, 25th, 75th and 90th percentiles and dots show individual datapoints.

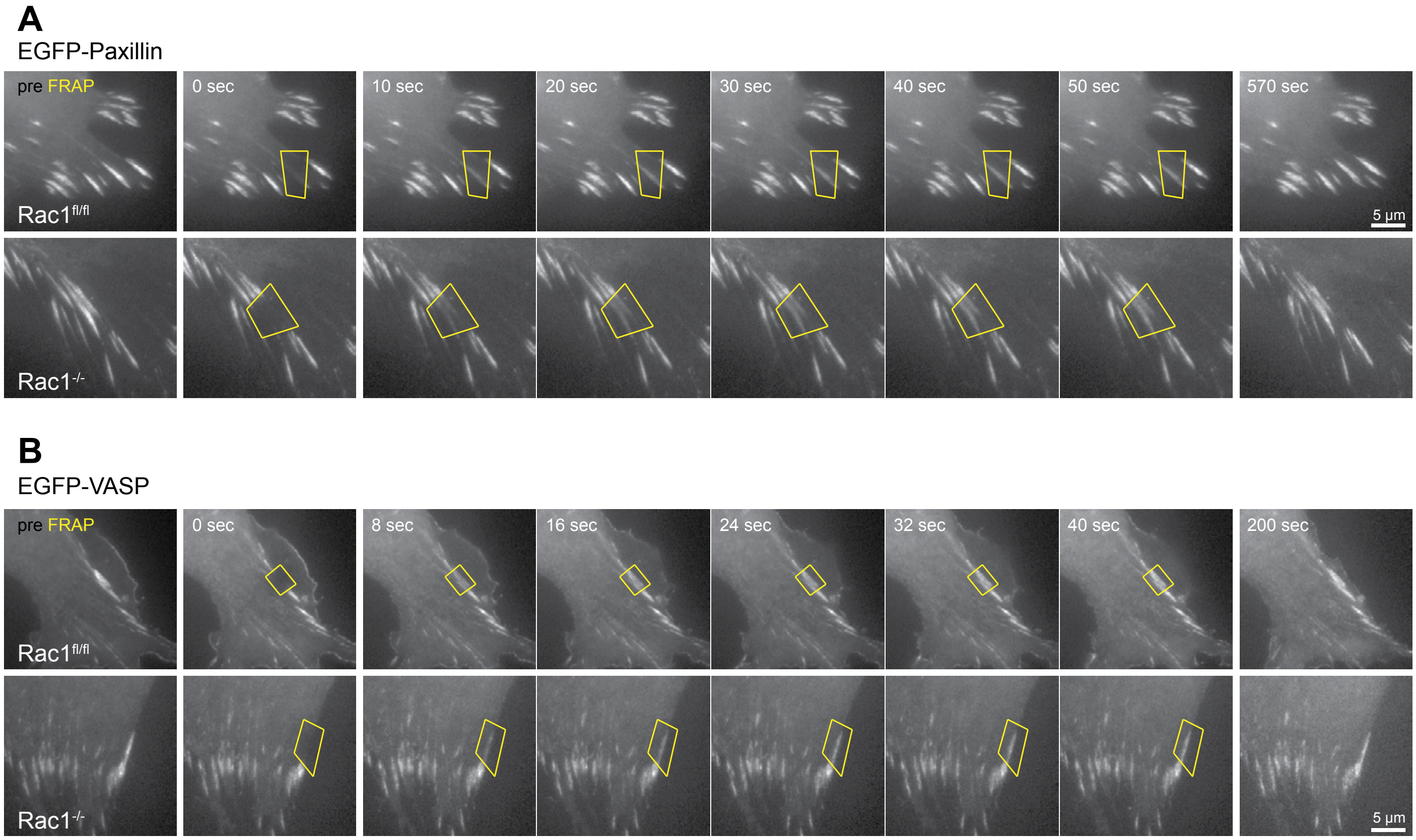

Fig. S9. Turnover of Paxillin and VASP in focal adhesions. (**A, B**) Representative frames of FRAP experiments of EGFP-tagged Paxillin (A) and VASP (B) in Rac1$^{fl/fl}$ and Rac1$^{-/-}$ cells, as indicated. Panels show localization of respective fluorescent component before (pre FRAP, left panel), immediately after bleaching (0 sec) and during fluorescence recovery at time points as indicated. Yellow polygons mark bleached areas.

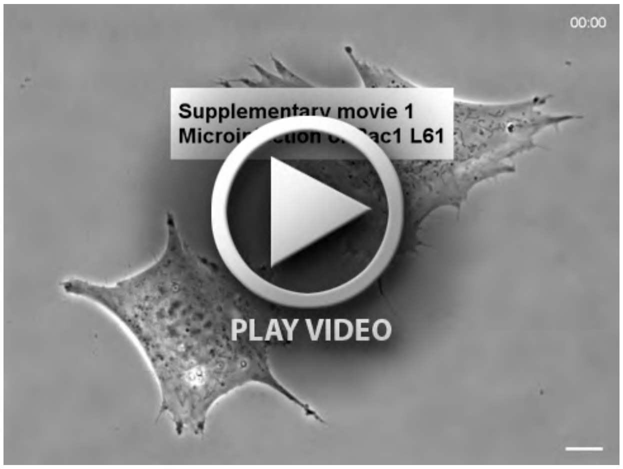

Movie 1. Instantaneous lamellipodia induction in Rac1$^{-/-}$ cells by microinjection of Rac1 L61. Phase contrast time lapse microscopy of Rac1$^{-/-}$ cells shows microinjection of constitutively active Rac1 (L61) protein. Time point of injection of left cell at 07:34 is also indicated with an arrow. Note immediate induction of lamellipodia around the entire cell periphery and concomitant loss of filopodia. The upper right cell remained uninjected and continued to protrude filopodia during continuous image acquisition. Elapsed time is in minutes:seconds, scale bar equals 10 µm.

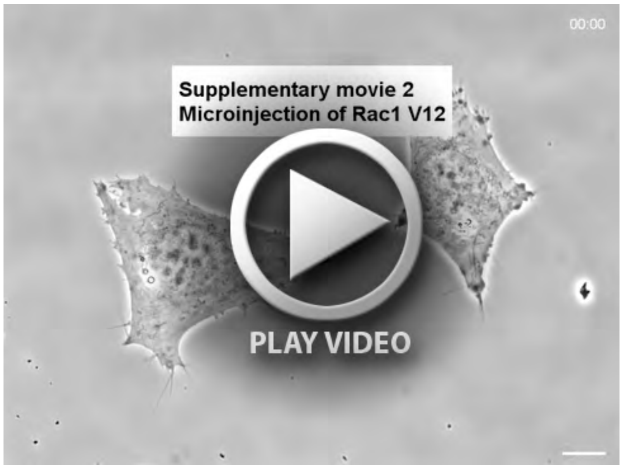

Movie 2. Instantaneous lamellipodia induction in Rac1$^{-/-}$ cells by microinjection of Rac1 V12. Phase contrast time lapse microscopy of Rac1$^{-/-}$ cells showing microinjection of constitutively active Rac1 (V12) at 04:09 into lower left cell. Note immediate induction of lamellipodia at the expense of filopodia. The non-injected cell continued to protrude multiple filopodia during entire experiment. Time is minutes:seconds, scale bar equals 10 µm.

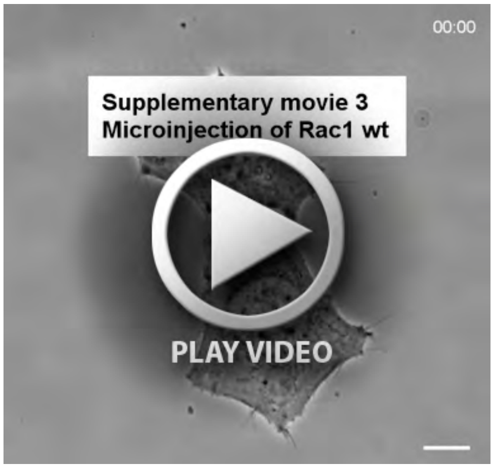

Movie 3. Instantaneous lamellipodia induction in Rac1$^{-/-}$ cells by microinjection of Rac1 wt. Phase contrast time lapse microscopy of Rac1$^{-/-}$ cells showing microinjection of one cell (as indicated) with wild type Rac1 protein. Again, lamellipodia are formed promptly after injection, and filopodia mostly lost. The uninjected cell continued protruding multiple filopodia. Time is given in minutes:seconds, scale bar represents 10 µm.

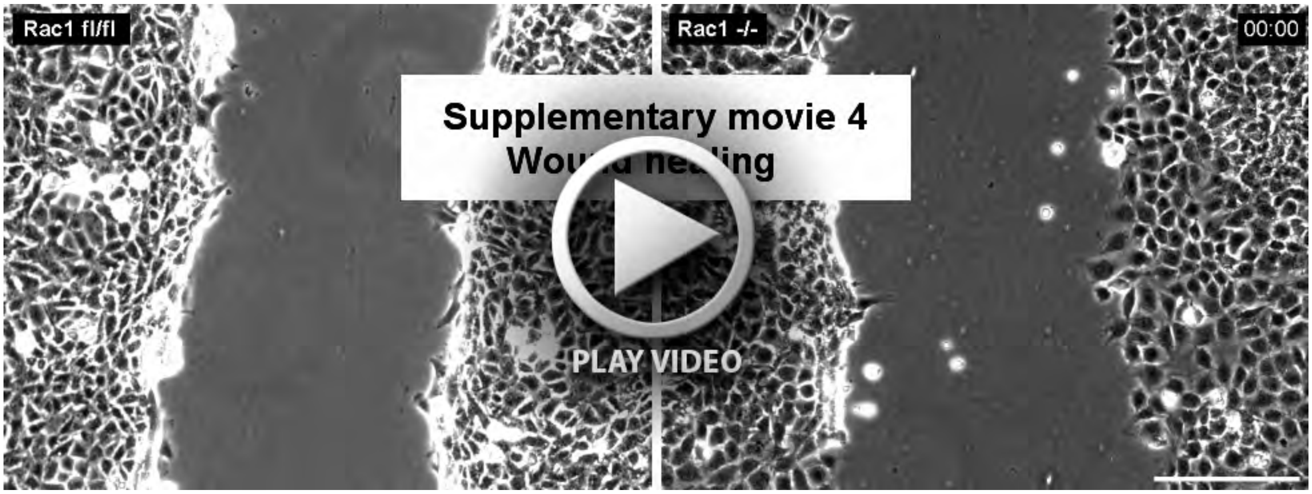

Movie 4. Wound healing assay of Rac1$^{fl/fl}$ and $^{-/-}$ fibroblasts. Phase contrast time lapse microscopy of Rac1$^{fl/fl}$ (left) and Rac1$^{-/-}$ cells (right) during wound healing. Elapsed time is displayed in hours:minutes, scale bar equals 200 µm.

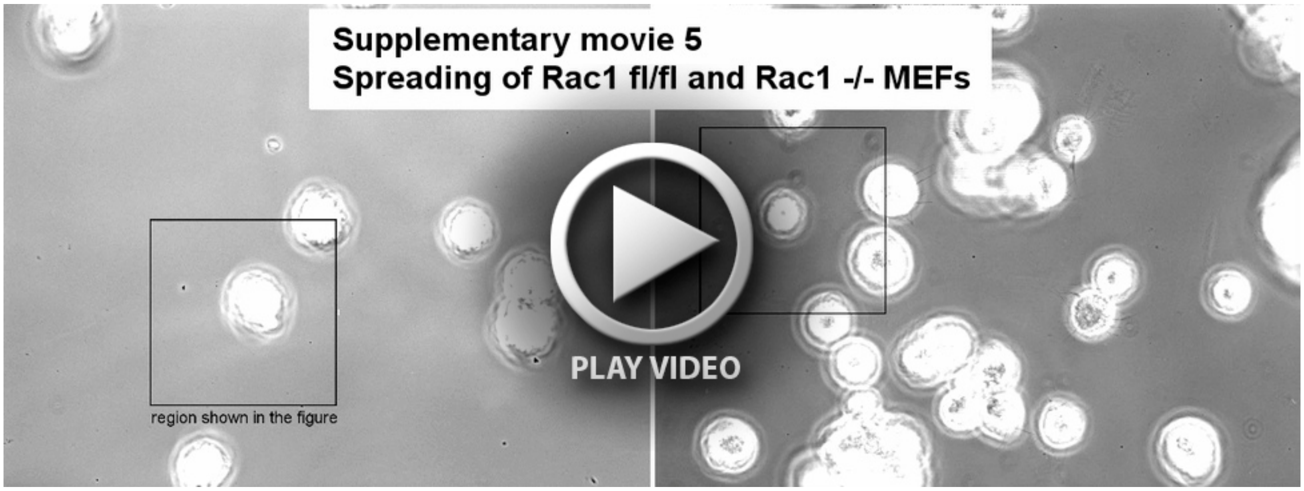

Movie 5. Rac1$^{-/-}$ fibroblasts have no spreading defect. Phase contrast time lapse microscopy of Rac1$^{fl/fl}$ (left) and Rac1$^{-/-}$ (right) cells during spreading. The regions shown in Figures 5G and 5H are marked with black squares. Note that while Rac1$^{fl/fl}$ cells spread by employing lamellipodia and ruffles, spreading of Rac1$^{-/-}$ fibroblasts is accompanied by formation of multiple filopodia. Elapsed time is in minutes:seconds, scale bar equals 20 µm.

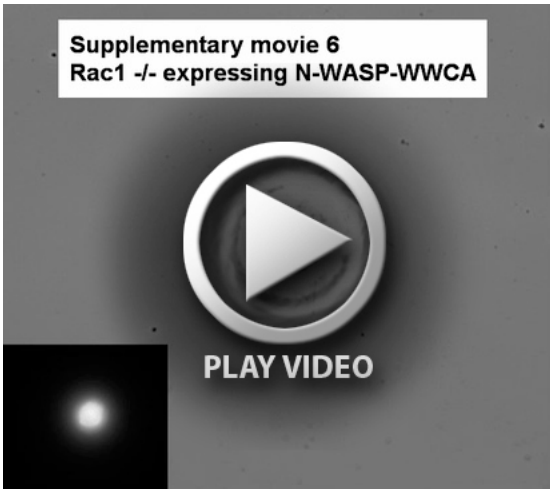

Movie 6. Additional Arp2/3 complex inhibition in Rac1$^{-/-}$ cells does not interfere with cell spreading. Phase contrast and epifluorescence (insert) time lapse movie of Rac1$^{-/-}$ cell ectopically expressing EGFP-N-WASP-WWCA. Spreading is indistinguishable from cells lacking WWCA expression (compare Supplementary Movie 5), and accompanied by strong formation of multiple filopodia. Elapsed time is given in minutes:seconds, scale bar equals 20 µm.

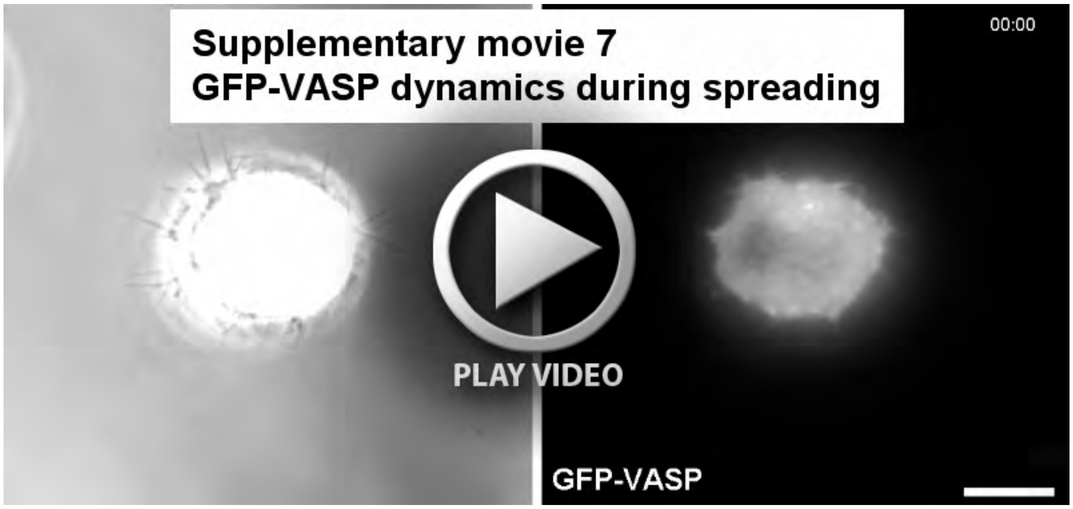

Movie 7. EGFP-VASP dynamics in Rac1$^{-/-}$ cell during spreading. Phase contrast (left) and epifluorescence (right) time lapse movie of an EGFP-VASP expressing Rac1$^{-/-}$ cell showing VASP dynamics during spreading. Note that VASP accumulates at the base of filopodia after their protrusion. EGFP-VASP intensity at filopodia tips is relatively weak as compared to focal adhesions. Note the apparent lack in this cell type of sharp, horizontal lines of VASP accumulation at the cell periphery, commonly marking the tips of protruding lamellipodia in the presence of Rac. Elapsed time is in minutes:seconds, scale bar, 10 µm.

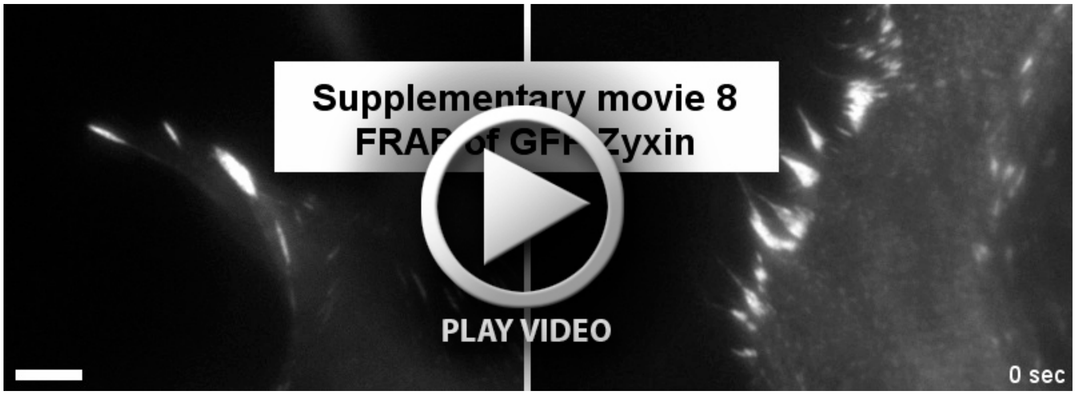

Movie 8. Representative examples of Zyxin turnover in focal adhesions as revealed by fluorescence recovery after photobleaching. Movies of Rac1$^{fl/fl}$ (left) and Rac1$^{-/-}$ (right) cells expressing EGFP-Zyxin. Cells were imaged every 2 seconds by epifluorescence microscopy before and after bleaching. Arrows point to bleached focal adhesions. Note similar recovery rates of Zyxin fluorescence in focal adhesions of Rac1$^{fl/fl}$ versus Rac1$^{-/-}$ cells. Movies are related to Fig. 9 A, B. Scale bar, 5 µm.

Carl, U. D., Pollmann, M., Orr, E., Gertlere, F. B., Chakraborty, T. and Wehland, J. (1999). Aromatic and basic residues within the EVH1 domain of VASP specify its interaction with proline-rich ligands. *Curr Biol* **9**, 715-718.

Lai, F. P., Szczodrak, M., Oelkers, J. M., Ladwein, M., Acconcia, F., Benesch, S., Auinger, S., Faix, J., Small, J. V., Polo, S. et al. (2009). Cortactin promotes migration and platelet-derived growth factor-induced actin reorganization by signaling to Rho-GTPases. *Mol Biol Cell* **20**, 3209-3223.

Lommel, S., Benesch, S., Rottner, K., Franz, T., Wehland, J. and Kuhn, R. (2001). Actin pedestal formation by enteropathogenic Escherichia coli and intracellular motility of Shigella flexneri are abolished in N-WASP-defective cells. *EMBO Rep* **2**, 850-857.

Monypenny, J., Zicha, D., Higashida, C., Oceguera-Yanez, F., Narumiya, S. and Watanabe, N. (2009). Cdc42 and Rac family GTPases regulate mode and speed but not direction of primary fibroblast migration during platelet-derived growth factor-dependent chemotaxis. *Mol Cell Biol* **29**, 2730-2747.

Rottner, K., Krause, M., Gimona, M., Small, J. V. and Wehland, J. (2001). Zyxin is not colocalized with vasodilator-stimulated phosphoprotein (VASP) at lamellipodial tips and exhibits different dynamics to vinculin, paxillin, and VASP in focal adhesions. *Mol Biol Cell* **12**, 3103-3113.

Supplementary Tables

Table S1. Plasmids employed in this study.

Plasmid name	Generation	Reference
pEGFP-VASP		(Carl et al., 1999)
pEGFP-N-WASP-WWCA		(Lommel et al., 2001)
pEGFP-Rac1		(Monypenny et al., 2009)
pEGFP-Rac2		(Monypenny et al., 2009)
pEGFP-Rac3		(Monypenny et al., 2009)
pEGFP-RhoG		(Monypenny et al., 2009)
pEGFP-Cdc42-L61		(Lai et al., 2009)
pRK5-myc-Rac1-L61		Alan Hall (Memorial Sloan-Kettering Cancer Center, New York, USA)
pRK5-myc-Rac1-N17		Alan Hall (Memorial Sloan-Kettering Cancer Center, New York, USA)
pGEX-2T-Rac1-L61		Alan Hall (Memorial Sloan-Kettering Cancer Center, New York, USA)
pRK5-myc-Cdc42-L61		Alan Hall (Memorial Sloan-Kettering Cancer Center, New York,

		USA)
pGEX-2T-Cdc42-L61		Alan Hall (Memorial Sloan-Kettering Cancer Center, New York, USA)
EGFP-Zyxin		(Rottner et al., 2001)
EGFP-Paxillin		(Rottner et al., 2001)
psiRNA-h7SKGFPzeo		Invivogen
pcDNA3.1-3xHA-Rac2-wt		Missouri S&T cDNA Resource Center
pcDNA3.1-3xHA-Rac2-L61	site directed mutagenesis (Quick change, Stratagene) on plasmid #16 using strand primer: 5'-TGGGACACTGCTGGGCTGGAGGACTACGACCGT	This work
pRK5-myc-Rac2-L61	PCR amplification of plasmid #17 using 5'-TTGGATCCCAGGCCATCAAGTGTGTGG-3' and 5'-TGAATTCTAGAGGAGGCTGCAGGC-3'; ligation into BamHI/EcoRI sites of the vector backbone of pRK5-myc-Rac1-L61	This work
pGEX-6P-2-Rac2-L61	subcloned from plamid #18 and ligated into BamHI/EcoRI sites of pGEX-6P-2	This work
pcDNA3.1-3xHA-Rac3-wt		Missouri S&T cDNA Resource Center
pcDNA3.1-3xHA-Rac3-L61	site directed mutagenesis (Quick change, Stratagene) on plasmid #20 using strand primer: 5'-GGACACAGCGGGTCTGGAGGACTACGATC-3'	This work
pRK5-myc-Rac3-L61	PCR amplification of plasmid #21 using 5'-TTGGATCCCAGGCCATCAAGTGCGTGG-3' and 5'-TGAATTCTAGAAGACGGTGCACTTCTTCC-3'; ligation into BamHI/EcoRI sites of the vector backbone of pRK5-myc-Rac1-L61	This work
pGEX-6P-1-Rac3-L61	PCR amplification of plasmid #21 using 5'-TTGGATCCCAGGCCATCAAGTGCGTGG-3' and 5'-AGACTCGAGCTAGAAGACGG-3'; ligation into BamHI/XhoI sites of pGEX-6P-1	This work

pcDNA3-3xmyc-RhoG-wt		Pontus Aspenstrom (Karolinska Institute, Stockholm, Sweden)
pcDNA3-3xmyc-RhoG-L61	site directed mutagenesis (Quickchange, Stratagene) on plasmid #24 using strand primer: 5'-TGGGACACTGCGGGCCTGGAGGAGTATGACCGC-3'	This work
pRK5-myc-RhoG-L61	PCR amplification of plasmid #25 using primers: 5'-TTGGATCCCAGAGCATCAAGTGCGTGG-3' and 5'-AGAATTCACAAGAGGATGCAGGACC-3'; ligation into BamHI/EcoRI sites of pRK5-myc-Rac1-L61	This work
pGEX-6P-3-RhoG-L61	PCR amplification of plasmid #25 using primers: 5'-TTGGATCCCAGAGCATCAAGTGCGTGG-3' and 5'-AGAATTCACAAGAGGATGCAGGACC-3'; ligation into BamHI/EcoRI sites of pGEX-6P-3	This work
pRK5-myc-Rac1-L61-deltaCAAX	PCR amplification of plasmid #8 using primers: 5'-CTGGGATCCCCGCAGGCC-3' and 5'-AAGTTGAATTCTCATTTTCTCTTCCTCTTCTTCACGG-3'; ligation into BamHI/EcoRI sites of pRK5-myc-Rac1-L61	This work

GTPase constructs mediating expression of myc- or GST-tagged Rac1, Rac2, Rac3 and RhoG were cloned with the same linker sequence. Following the 5' BamHI restriction site, ATG was deleted such that BamHI site is followed by the second codon in each case. All generated constructs were sequence verified.

Table S2. Antibodies, reagents and treatments used in this study.

	Reference	Specification of treatments
Mouse anti-Rac1 termed anti-Rac1/3 here (23A8)	Biomol	
Mouse anti-myc (9E10)	Abcam	
Mouse anti-Vinculin (clone hVIN-1)	Sigma	
Mouse RhoG antibody (clone 1F3 B3 E5)	Millipore	
Mouse anti-alpha-Tubulin (clone 3A2)	Synaptic Systems	
Mouse anti-GFP (clone 101G4)	Synaptic Systems	
Monoclonal Rac2 antibody 273-75-1	This study	
Rabbit Sra-1B antibody	Steffen et al., 2004	
Mouse anti-Abi	Giorgio Scita (IFOM, Milan, Italy)	
Alexa-594 coupled to goat anti-mouse	Invitrogen	
Alexa-594 coupled to goat anti-rabbit	Invitrogen	
Alexa-488 coupled to phalloidin	Invitrogen	
Peroxidase coupled to goat anti-mouse	Dianova	
Peroxidase coupled to goat anti-rabbit	Dianova	
PDGF (PDGF-BB)	Sigma	10 ng/ml for 5 minutes after o/n starvation in DMEM
EGF	Sigma	100 ng/ml for 5 minutes after o/n starvation in DMEM
HGF	Sigma	20 ng/ml for 5 minutes after o/n starvation in DMEM
Rho Kinase inhibitor Y27632	Biozol	20 µM, added 1 hour prior to wound scratching

Table S3. Co-efficient values corresponding to curve fit equations provided in Fig. 9D, F, H.

		y(0)	a	b	c	d
Zyxin	Rac1 $^{fl/fl}$	0.0221	0.3712	0.0495	n.a.	n.a.
Zyxin	Rac1 $^{-/-}$	0,0361	0,5187	0.0463	n.a.	n.a.
Paxillin	Rac1 $^{fl/fl}$	n.a.	0,2265	0,0624	0,1502	0.0038
Paxillin	Rac1 $^{-/-}$	n.a.	0,1924	0,0908	0,3067	0,0071
VASP	Rac1 $^{fl/fl}$	n.a.	0,2989	0.1998	0,1509	0.020
VASP	Rac1 $^{-/-}$	n.a.	0,1310	0,3253	0,4480	0,0505
